# Site-Selective Oxide Rearrangement in a Tandem Metal–Metal Oxide Catalyst Improves Selectivity in Oxidative Dehydrogenation of Propane

**DOI:** 10.1021/jacs.5c13571

**Published:** 2025-10-28

**Authors:** Snehitha Srirangam, Siddharth Deshpande

**Affiliations:** Department of Chemical and Sustainability Engineering, 6927 University of Rochester, Rochester, New York 14627, United States

## Abstract

Tandem metal–metal oxide catalysts, where metallic and metal oxide active sites work synergistically to drive complex chemistries, have been shown to improve the catalyst stability, activity, and selectivity. Although experimental techniques have probed the active site structure of such catalysts, the key atomic features that drive structure evolution under synthesis and reaction conditions remain poorly understood. Here, we develop a computational framework to elucidate the chemical, geometric, and stoichiometric features of the tandem overcoated catalyst, Pt-InO_*x*_H_*y*_, in driving the Oxidative Propane Dehydrogenation (ODHP) reaction, integrating propane dehydrogenation (PDH) and Selective Hydrogen Combustion (SHC). Exploration of the chemical space of stable InO_*x*_H_*y*_ phases on Pt relevant to the experimental conditions reveals that the pore formation of the ALD-deposited InO_*x*_H_*y*_ catalyst results from the oxide destabilization on well-coordinated Pt-terrace sites and preferential decoration around under-coordinated Pt-step sites. Reaction mechanistic analysis of the Pt-InO_*x*_H_*y*_ catalyst reveals a dual-site mechanism for SHC, where O* activates on Pt and subsequently forms OH* at the InO_*x*_H_*y*_ sites, facilitating water formation and controlling overoxidation. Further, due to passivation of the under-coordinated Pt-step sites, the Pt-InO_*x*_H_*y*_ surface exhibits similar PDH activity as that on a well-coordinated Pt-terrace surface, in addition to enhanced stability by destabilizing deep-dehydrogenated intermediates. These insights establish a structure–performance relationship of the Pt-InO_*x*_H_*y*_ catalyst for ODHP chemistry, with key features being the extent of reducibility of the metal oxide and the sensitivity of the oxide structure to the oxygen chemical potential. This framework can be extended to other metal–metal oxide systems and complex reactions to develop next-generation tandem catalysts.

## Graphical abstract

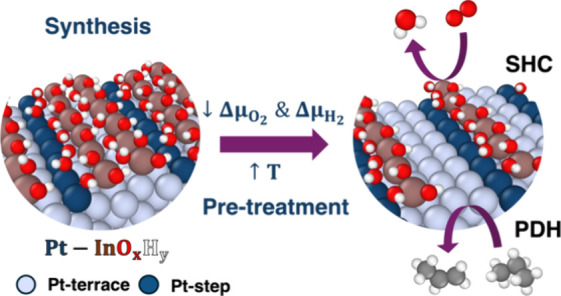

## Introduction

Heterogeneous tandem catalysts contain two or more distinct catalytic sites that synergistically drive key reaction steps, yielding high selectivity/activity/stability often difficult to obtain using individual sites alone.^1–5^ One subclass of such catalysts is metal–metal oxide catalysts, where the metal and metal oxide active sites work in tandem, while both sites remain exposed during the reaction. These catalysts have demonstrated enhanced performance in driving key complex reaction chemistries, including alkane dehydrogenation, biomass valorization, and methanol steam reforming, among others.^6–9^ Advanced characterization techniques have provided insights into understanding the active site behavior of such metal–metal oxide catalysts.^6,10–13^ Based on these studies, the enhanced catalytic performance is attributed to various phenomena,^14^ with structural rearrangement of the metal and metal oxide phases under reaction conditions being an important factor influencing catalytic performance.^15–17^ However, the chemical and geometric features of the active phases that control these structural transformations and reaction kinetics at the interface during the synthesis and reaction remain poorly understood. This lack of understanding leads to relying on trial-and-error approaches to determine optimal metal–metal oxide (M_1_ – M_2_ O_*x*_ H_*y*_) catalysts. Novel methods that can overcome these challenges by elucidating the governing atomic features of the M_1_ – M_2_ O_*x*_ H_*y*_ active sites are hence desired to conduct a systematic physics-driven search for optimal catalysts.

First-principles methods, such as Density Functional Theory (DFT), can reveal the electronic and geometric features that govern the structure, stability, and reactivity of M_1_ – M_2_ O_*x*_ H_*y*_ catalysts. Recent computational approaches have predicted the catalyst structures at the M_1_ – M_2_ O_*x*_ H_*y*_ interface by identifying low-energy surface oxide structures on a metal at reaction conditions.^18–20^ For example, Kempen and Andersen identified the stable oxide geometries of Zn_*y*_O_*x*_ and In_*y*_O_*x*_ on fcc(111) metal surfaces using a global optimization workflow.^20^ Kumari *et al.* identified low-energy configurations of different ligands such as formate, oxygen, and hydroxyls on ZrO_*x*_ in a ZrO_2_/Cu­(111) catalyst under CO_2_ hydrogenation conditions using the grand canonical basin hopping algorithm.^19^ Although significant advances have been made, incorporating the diverse atomic features that accurately represent the active site structure under in situ experimental conditions remains a key challenge, often called the Material Gap.^21^ A key step in bridging this gap requires consideration of additional structural and chemical features in the atomic models. These features include the presence of potential defects on the metal, varying oxide stoichiometries in the presence of oxidizing and reducing environments, considering the variable coverage of oxide as a function of reaction conditions, and the presence of functional groups such as OH* on the oxide surface. However, considering the above-mentioned structural and chemical features to construct accurate atomic models is hindered due to the vast configuration space (sampling challenge). This work addresses this sampling challenge by adopting an algorithmic framework for systematically investigating the metal–metal oxide interface to identify M_1_ – M_2_ O_*x*_ H_*y*_ active site structures under experimental conditions. We utilize the framework to study the important case of Oxidative Propane Dehydrogenation (ODHP) reaction on the tandem overcoated In_2_O_3_-Pt/Al_2_O_3_ catalyst.^7^

ODHP is an alternative process to propane dehydrogenation (PDH) where an oxidant such as O_2_ (often referred to as O_2_ assisted-ODHP or O_2_-ODHP) is added to make the overall reaction exothermic and overcome its equilibrium limitations.^22^ However, achieving high selectivity in O_2_-ODHP is a challenge due to multiple side reactions, including combustion, cracking, and overoxidation.^23^ Recently, using a tandem ODHP reaction that integrates PDH and Selective Hydrogen Combustion (SHC), Yan *et al.* demonstrated the In_2_O_3_-Pt/Al_2_O_3_ catalyst to successfully overcome these challenges.^7^ The performance of this catalyst was proposed to be intrinsically linked to the local structure of the indium oxide (InO_*x*_) overcoat at the metal–metal oxide interface. Specifically, the catalyst is shown to undergo rearrangement, exposing both metal and metal oxide active sites during the reaction, which tightly couples the reactions for direct transfer of intermediates between the catalyst domains (Pt and In_2_O_3_). Nevertheless, the nature of the underlying atomic structure and features that dictate these structural rearrangements and their subsequent effect on product selectivity remain unknown, hence hindering a physics-driven search for optimal tandem ODHP catalysts. Our proposed algorithmic framework, for the first time, elucidates the influence of different geometric, stoichiometric, and chemical features of the oxide and metal phases in selectively driving the ODHP reaction, overcoming this challenge.

The computational framework developed in this work couples first-principles-based Density Functional Theory (DFT) with a unique data-driven algorithm and successfully tackles the sampling challenge associated with modeling complex reactions on intricate M_1_ – M_2_ O_*x*_ H_*y*_ catalysts. The framework builds upon the work by Deshpande and Vlachos^24^ and now incorporates a systematic consideration of Brønsted acid sites and consideration of mixed O*/OH* functional groups to study the growth and coverage of indium oxide (In_2_O_3_) on the platinum (Pt) surface in the presence of defects under ODHP conditions. Our approach utilizes a modified graph-theory-based approach^24^ to identify all unique, stable configurations of indium oxide (InO_*x*_H_*y*_) on a defective Pt surface. These stable structures are used to construct a thermodynamic surface phase diagram, which for the first time elucidates the dynamic reconstruction of the surface oxide active sites from synthesis to catalytic conditions. The dominant surface oxide phase, identified as the stable structure under the reaction conditions, is used to study the thermodynamics and kinetics of the ODHP reaction. Through our analysis, we highlight the key atomic features that drive selective and stable ODHP on the Pt-InO_*x*_H_*y*_ catalyst, thus establishing a structure–performance relationship between the tandem metal–metal oxide catalyst and oxidative alkane dehydrogenation chemistry. The identified relationship, along with the algorithmic framework, now becomes a stepping stone to study other important complex chemistries in the important class of M_1_ – M_2_ O_*x*_ H_*y*_ catalysts.

## Computational Methods

Periodic density functional theory (DFT) calculations are performed using the Vienna *Ab initio* Simulation Package (VASP),^25^ where the Kohn–Sham equations are solved using the Perdew, Burke, and Ernzerhof functional (PBE)^26^ self-consistently with the Projector Augmented Wave (PAW) method.^27^ To model the Pt(111) surface, a 4 × 4 × 4 unit cell is utilized, while for Pt(322), a 4 × 5 × 4 unit cell is chosen, with an optimized lattice constant of 3.97 Å (Supporting Information, 7.8). A vacuum spacing of at least 11 Å on each side of the slab is considered in the *z*-direction, with the bottom layer of the slab constrained. A planewave energy cutoff of 400 eV is used with a 3 × 3 × 1 k-point set for all surfaces. The partial electronic occupancies are determined according to a Methfessel–Paxton scheme^28^ with an energy smearing of 0.2 eV. The structures are relaxed until the Hellmann–Feynman forces on the atoms are less than 0.05 eV/Å. The dipole and spin corrections have a negligible effect on the energy of the relaxed system and are therefore not considered (Supporting Information, 7.9).

The indium oxide (InO_*x*_H_*y*_) structure is modeled on the Pt(322) surface, which provides realistic surface characteristics by including four layers of well-coordinated terrace sites and one layer of under-coordinated step edge sites. The binding energy of InO_*x*_H_*y*_ on the Pt surface is evaluated as the formation energy of InO_*x*_H_*y*_ on Pt(322) (*E*_form_(In_*x*_O_*y*_H_*z*_)) referenced to the bulk indium oxide (In_2_O_3_) as shown in eq 1. Here, *E*_DFT_(Pt – In_*x*_O_*y*_H_*z*_) represents the potential energy of InO_*x*_H_*y*_ on Pt(322), *E*_DFT_(Pt) is the potential energy of Pt(322), and *E*_DFT_(In_2_O_3_) is the potential energy of cubic In_2_O_3_ structure adopted from The Materials Project.^29^

$$E_{\mathrm{form}}({\mathrm{In}}_{x}O_{y}H_{z})=E_{\mathrm{DFT}}(\mathrm{Pt}-{\mathrm{In}}_{x}O_{y}H_{z})-E_{\mathrm{DFT}}(\mathrm{Pt})-E_{\mathrm{DFT}}({\mathrm{In}}_{2}O_{3})-(x-2)\times \mu_{\mathrm{In}}-(y-3)\times \mu_{O}-(z)\times \mu_{H}+\mathrm{ZPE}(N_{{\mathrm{OH}}^{*}},N_{H_{2}O^{*}},N_{O^{*}})$$ 1

μ_In_, μ_O_, and μ_H_ are the chemical potentials of indium, oxygen, and hydrogen, respectively. ZPE­(*N*_OH*_, *N*_H_2_O*_, and *N*_O*_) corresponds to the sum of zero-point energy (ZPE) contributions of OH*, H_2_O*, and O* adsorbates on Pt-InO_*x*_H_*y*_, respectively. The zero-point energies are calculated based on harmonic vibrational states (Supporting Information, 1.1). The entropies of InO_*x*_H_*y*_ and bulk In_2_O_3_ structures are assumed to be similar and will cancel out. The adsorbed oxygen and hydrogen are approximated to be in quasi-equilibrium with their respective gas-phase components. The chemical potential of indium can be estimated from bulk In_2_O_3_ as a reference, as shown in eq 2, assuming the source as bulk indium oxide (In_2_ *O*_3_(*s*)).

$$\mu_{\mathrm{In}}=E_{\mathrm{DFT}}({\mathrm{In}}_{2}O_{3})-\left(\frac{3}{2}\right)\mu_{O}$$ 2

Under the reaction conditions, the chemical potentials of oxygen gas (μ_O_) and hydrogen gas (μ_H_) are evaluated using their standard chemical potentials at the reaction temperature and standard pressure (*p* ^0^). A pressure correction term is then applied based on the partial pressure (*p*_i_) of the gases relevant to the experimental conditions. The chemical potential of oxygen gas (O_2_) is estimated at 723 K (T) using the free energy of the oxygen reduction reaction,^30^ and the chemical potentials of hydrogen gas (H_2_) and water­(H_2_O) based on eq 3. The computation of the chemical potential of gas-phase components at 723 K and *p* ^0^ is further discussed in the Supporting Information, 1.1. The partial pressures of propane (*P*_C_3_H_8__) and oxygen (*P*_O_2__) are considered as 10 and 5 kPa, respectively, in line with the experimental conditions used in the ODHP reaction.^7^ Assuming negligible gas-phase combustion during the reaction conditions, the chemical potential of oxygen gas is evaluated at a pressure of 5 kPa by adding the pressure correction to the chemical potential at 723 K and standard pressure of 1 bar, as shown in eq 4. To understand the catalyst structure under varying gas pressures of O_2_, a range of oxygen chemical potentials is considered. These chemical potentials are referenced to oxygen gas at 5 kPa. The chemical potential of H_2_ gas is computed under steady-state conditions at 1.32 kPa. The choice of the chemical potential of H_2_ gas is described in the Supporting Information, 1.2.

$$\mu_{O}=\frac{1}{2}\times \mu_{O_{2}(g)}=\mu_{H_{2}O}-\mu_{H_{2}}+2\times 1.12$$ 3

$$\Delta \mu_{i}(T,p_{i})=\Delta \mu_{i}(T,p^{0})+k_{B}T\ln\frac{p_{i}}{p^{0}}$$ 4

Stable InO_*x*_H_*y*_ structures at different coverages on Pt(322) are initially estimated by calculating the formation energies using bulk In_2_O_3_ (μ_In_^′^) chemical potential, and the hydrogen (μ_H(gas)_^′^) and oxygen (μ_O(gas)_^′^) chemical potentials relevant to experimental conditions at 723 K (Supporting Information, 1.2). A phase diagram, as a function of the chemical potentials of hydrogen and oxygen gases, and In chemical potential referenced to the bulk In_2_O_3_, is then constructed to predict stable structures ranging from synthesis and pretreatment to reaction conditions.

Further, the formation energies of key ODHP intermediates are evaluated on Pt-InO_*x*_H_*y*_, Pt(111), and Pt(322) catalyst surfaces to understand the adsorption thermodynamics and kinetics of the ODHP reaction. These three surfaces are chosen to systematically understand the role of well-coordinated and under-coordinated (terrace vs steps) metal sites, along with InO_*x*_H_*y*_, in driving the PDH reaction. The key reaction intermediates for PDH mechanistic analysis are chosen based on a previous theoretical analysis.^31^ The formation energy of an intermediate is computed relative to the gas-phase energy of propane on the Pt-InO_*x*_H_*y*_ catalyst using eq 5.

$$E_{\mathrm{ads}}(C_{3}H_{x})=E_{\mathrm{DFT}}(C_{3}H_{x}-B)-E_{\mathrm{DFT}}(B)-E_{C_{3}H_{8}}+(8-x)\times \mu_{H}$$ 5

$$G_{\mathrm{ads}}=E_{\mathrm{ads}}+\mathrm{ZP}E_{\mathrm{ads}}-{\mathrm{TS}}_{\mathrm{ads}}$$ 6

Here, *E*_ads_(C_3_H_*x*_) is the formation energy of C_3_H_*x*_ relative to the energy of propane denoted as *E*_C_3_H_8__ and *E*_DFT_(C_3_H_*x*_ − **B**) is the potential energy of C_3_H_*x*_ adsorbed on a surface **B** corresponding to Pt-InO_*x*_H_*y*_, Pt(111), or Pt(322) surfaces, respectively.

The standard free energies for all adsorbates are calculated at 723 K based on eq 6. The zero-point energies are calculated based on harmonic vibrational states, and the standard state entropy corrections are calculated using the harmonic oscillator approximation.^31^ For a physisorbed propane* intermediate, entropy corrections are calculated as described by Seemakurthi *et al.*,^31^ accounting for hindered translator and rotor modes.^32,33^ The standard free energies of gas-phase components such as propane and propylene are calculated using ASE,^33^ based on ideal gas assumption at 723 K. Activation barriers for key elementary steps are calculated using the Climbing Image Nudged Elastic Band (CI-NEB) method.^34^ Eight images are generated between the initial and final states using the Image Dependent Pair Potential (IDPP),^35^ and the CI-NEB calculations are conducted. The transition state (TS) for propylene gas desorption is computed using the 2D-ideal gas assumption.^31,36^ TS entropies of adsorbates are approximated by the initial and final state entropies, depending on whether the TS is closer to the initial or final states.^31^

## Results and Discussion

To elucidate the role of the active site structure of Pt-InO_*x*_H_*y*_ in driving ODHP, structural analysis is initially performed. An algorithmic approach is utilized to explore the diverse range of possible InO_*x*_H_*y*_ configurations on a Pt surface. Building upon this, an *ab initio* thermodynamic phase diagram is constructed to understand the formation of InO_*x*_H_*y*_ on Pt, mapping the evolution of the InO_*x*_H_*y*_ structure on Pt from synthesis to that under reaction conditions. The latter part of the paper focuses on elucidating the role of a stable InO_*x*_H_*y*_ structure in driving the SHC reaction by exploring the oxygen adsorption and reduction thermodynamics on the catalyst. Following this, a thermodynamic and kinetic analysis is presented to understand PDH on the Pt-InO_*x*_H_*y*_ catalyst and discern the influence of the InO_*x*_ overlayer in selectively driving the PDH chemistry.

### Algorithmic Approach for InO_*x*_H_*y*_ Structure Search

Predicting the intricate structure of a metal oxide overlayer on a metal is challenging due to the vast number of possible configurations arising from varying coverage, stoichiometry, oxidation states, the possible formation of Brønsted acid sites, and the presence of defects on the underlying metal. These complexities hinder our understanding of the nature of the active site structure and its subsequent role in driving catalytic reactions. Hence, to overcome these sampling challenges, a systematic and data-efficient approach is required. Herein, the structure of InO_*x*_H_*y*_ on a defective Pt(322) surface is modeled by utilizing a unique workflow that integrates the modified SurfGraph algorithm with a formation energy-based evolutionary algorithm,^24^ as shown in Figure 1a.

**Figure 1:**
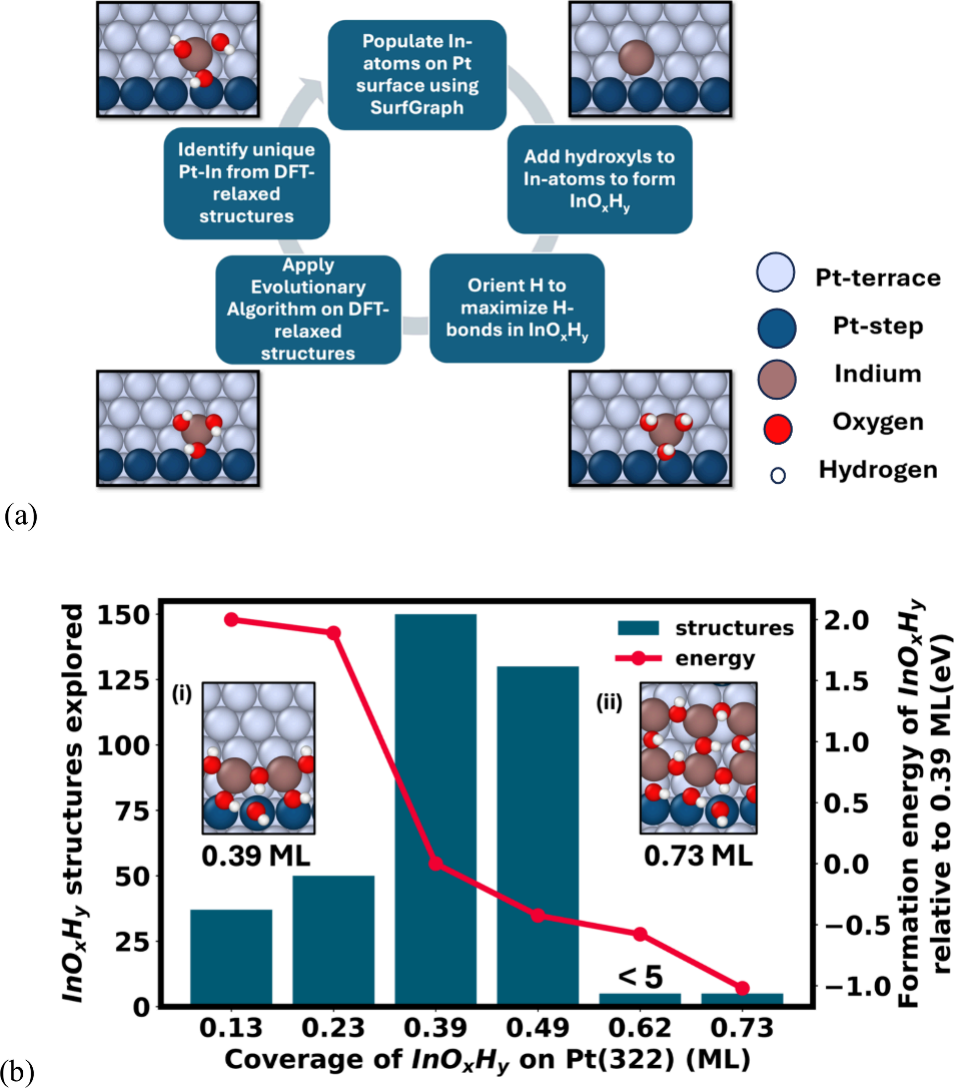
(a) Schematic of the workflow. In atoms are placed using a modified SurfGraph, followed by the addition of hydroxyl groups to form InO_*x*_H_*y*_. Structures with maximum H-bond distribution are retained, and H atoms are oriented based on H-bond donor–acceptor distances. (b) Number of structures explored at each InO_*x*_ coverage on Pt(322) and the formation energy of the most stable InO_*x*_H_*y*_ structure at each coverage as a function of the most stable structure at 0.39 ML. Insets (i) and (ii) show the most stable InO_*x*_H_*y*_ structures on Pt(322) at 0.39 and 0.73 ML, respectively. The formation energy is calculated using μ_In_^′^ with respect to bulk In_2_O_3_, μ_H(gas)_^′^ with respect to the partial pressure of H_2_ at the reactor output of 1.32 kPa, and μ_O(gas)_^′^ with respect to partial pressure of O_2_ at a reactor input of 5 kPa.

The workflow initiates with populating Pt(322) with unique starting positions of indium (In) atoms on Pt using SurfGraph^37^ (Step 1). In Step 2, each In atom is coordinated with three oxygen (O) atoms, as hydroxyl (OH) groups, mimicking the oxidation state of In present in the bulk. The choice of using OH* stems from a systematic investigation of the position and chemical nature of O atoms within the InO_*x*_H_*y*_ framework, which revealed that they are stable in the form of OH* groups (Supporting Information, 2.2), owing to the high proton affinity of surface oxygen atoms on In_2_O_3_.^38^ This stability holds within a specific range of chemical potentials of the hydrogen and oxygen gases, as discussed in subsequent sections. In Step 3, a directional graph-based approach is employed to identify stable configurations with maximized hydrogen bonding distribution.^39^ In Step 4, the structures are optimized using DFT, and the stable structures with formation energies within a cutoff are screened using the formation energy-based evolutionary algorithm.^24^ For any given In coverage consisting of *n* In atoms on the surface, the formation energy is evaluated by determining the difference in binding energies of the current structure and the most stable structure at the preceding coverage with (*n* – 1) In atoms. In Step 5, unique InO_*x*_H_*y*_ stable configurations are segregated using the modified SurfGraph algorithm by removing OH* groups and identifying unique Pt–In relaxed structures. These unique Pt–In structures are mapped back to the InO_*x*_H_*y*_ configurations to obtain a stable set of configurations at the desired coverage. The unique Pt–In relaxed structures are then used as a basis for the subsequent enumeration, where additional Indium atoms are populated based on the modified SurfGraph algorithm. This cycle is repeated until saturated InO_*x*_H_*y*_ coverage on the stepped Pt(322) surface is obtained. This approach allows for the determination of the InO_*x*_H_*y*_ overlayer structure as a function of coverage on the stepped Pt surface, providing insights into the key atomic interactions and geometric properties that play a role in stabilizing Pt-InO_*x*_H_*y*_ catalysts.

The results from the systematic exploration of InO_*x*_H_*y*_ configurations as a function of In coverage are presented in Figure 1b. With the increase in In coverage on Pt, the number of possible InO_*x*_H_*y*_ configurations increases substantially due to multiple orientations of InO_*x*_H_*y*_ structures and their interactions with various types of Pt sites. Importantly, by utilizing the computational workflow described in Figure 1a, the number of DFT simulations required to identify stable InO_*x*_H_*y*_ structures was systematically reduced from O­(10^4^) to O­(10^2^) (Supporting Information, 2.1). Through the structural exploration, it is revealed that the InO_*x*_H_*y*_ structures exhibit favorable interactions with the Pt-step sites compared to those lacking such interactions (Supporting Information, 2.3). The enhanced stability arises from strong binding between In and stepped Pt atoms, and the formation of hydrogen bonding networks through OH* groups that bridge In and Pt-step sites, as shown in insets (i) and (ii) in Figure 1b. As shown in Figure 1b inset (i), even at a modest coverage of 0.39 ML, the Pt-step edge becomes saturated with InO_*x*_H_*y*_, forming a continuous chain of hydrogen-bonded OH* groups. Utilizing these insights on Pt-InO_*x*_H_*y*_ binding, we adopted the assumption that for higher coverages beyond 0.5 ML, the step edges remain saturated with InO_*x*_H_*y*_ to maintain the stabilizing interactions between In and the Pt-step. This assumption allowed for efficient exploration of higher coverages beyond 0.5 ML as shown in Figure 1b. Following this approach, structures with higher In coverages revealed ring-like structures on the terrace sites coupled with chain-like arrangement preserved at the Pt-step sites (Supporting Information, 2.3). At the saturation coverage estimated at 0.73 ML, the InO_*x*_H_*y*_ phase occupies most of the Pt sites including the terrace and step sites with each In atom surrounded by at least three OH* groups as shown in the right inset in Figure 1b. The resulting stable structures across different In coverages exhibit stoichiometries of In_p_(OH)_2*p*+1_ and In_p_(OH)_2*p*+2_ above a coverage of 0.2 ML with each In atom coordinating with three to four OH* groups where *p* represents the number of InO_*x*_H_*y*_ units. The red line in Figure 1b represents the formation energy trends of the stable hydroxylated InO_*x*_H_*y*_ structures at each coverage under experimental conditions of μ_H(gas)_^′^ at 1.32 kPa of H_2_ and μ_O(gas)_^′^ at 5 kPa of O_2_ with reference to the energy of the most stable InO_*x*_ structure at 0.39 ML coverage on Pt. As shown in Figure 1b, the formation energy of InO_*x*_H_*y*_ decreases with an increase in its coverage on Pt(322), depicting an increased binding strength. However, this holds true within a certain range of chemical potentials of hydrogen and oxygen gases, as discussed in subsequent sections. Further, by utilizing the stable hydroxylated InO_*x*_ structures, we incorporated an additional framework to identify structures that might be stable under conditions away from those of the reactor. The procedure involves systematically varying both O and H coverages in the subset of InO_*x*_H_*y*_ structures identified through the algorithm presented in Figure 1a (Supporting Information, 2.5), resulting in the generation of active site structures with varying concentrations of the O/OH groups. These candidate structures are then DFT relaxed and compared for stability as a function of the O_2_ and H_2_ chemical potentials. For this study, the framework is applied to the fully hydroxylated InO_*x*_H_*y*_ structure at 0.39 ML coverage to generate possible partially hydroxylated structures with different stoichiometries of O and H atoms, which we now refer to as O and H concentrations in the InO_*x*_H_*y*_ structure. The choice of using 0.39 ML coverage stems from the dominance of this specific InO_*x*_ phase after the pretreatment conditions, as will be discussed further. Through this analysis, a library of stable atomic InO_*x*_H_*y*_ structures at different In coverages on Pt is now constructed. This library now provides the basis for understanding the structure evolution of the Pt-InO_*x*_H_*y*_ catalyst during synthesis, pretreatment, and reaction conditions, as discussed in the next section.

### Thermodynamic Phase Diagram of the InO_*x*_H_*y*_ Overlayer on the Pt(322) Surface

Elucidating the metal oxide structure during synthesis, pretreatment, and reaction conditions is crucial in understanding the role of the metal–metal oxide interface in driving the ODHP reaction. First-principles-based surface phase diagrams have been used to predict the structures of surface oxides under experimental conditions.^18,40,41^ These diagrams map thermodynamically favorable structures across varying chemical environments and chemical potentials, providing a systematic approach for the prediction of the evolution of the InO_*x*_H_*y*_ structure from synthesis through reaction conditions. Using the stable InO_*x*_H_*y*_ structures predicted at different coverages on Pt(322), a thermodynamic surface phase diagram is constructed as a function of the chemical potentials of gas phase O_2_ and H_2_ (μ_O_=1/2μ_O__2_, μ_H_=1/2μ_H_2__). μ_O_ and μ_H_ are varied to mimic different synthesis and pretreatment conditions, as shown in Figure 2a. A range of chemical potentials of Δμ_i(gas)_ is considered for oxygen and hydrogen gases, where Δμ_i(gas)_ is equal to (μ_i(gas)_ – μ_i(gas)_^′^). Here, μ_i(gas)_^′^ refers to chemical potentials of the O_2_ and H_2_ gases corresponding to the O_2_ reactor input of 5 kPa and the H_2_ reactor output of 1.32 kPa under the steady state (Supporting Information, 1.2), respectively, at a temperature of 723 K. μ_i(gas)_ corresponds to the chemical potentials of the O_2_ and H_2_ gases inside the reactor. A decrease in Δμ_i(gas)_ corresponds to a decrease in the partial pressures of the O_2_ and H_2_ gases in the reactor under experimental conditions. Stable structures are mapped across different chemical potentials using formation energies computed using eq 1. Figure 2b–g shows the stable structures corresponding to each contour region in the surface phase diagram.

**Figure 2:**
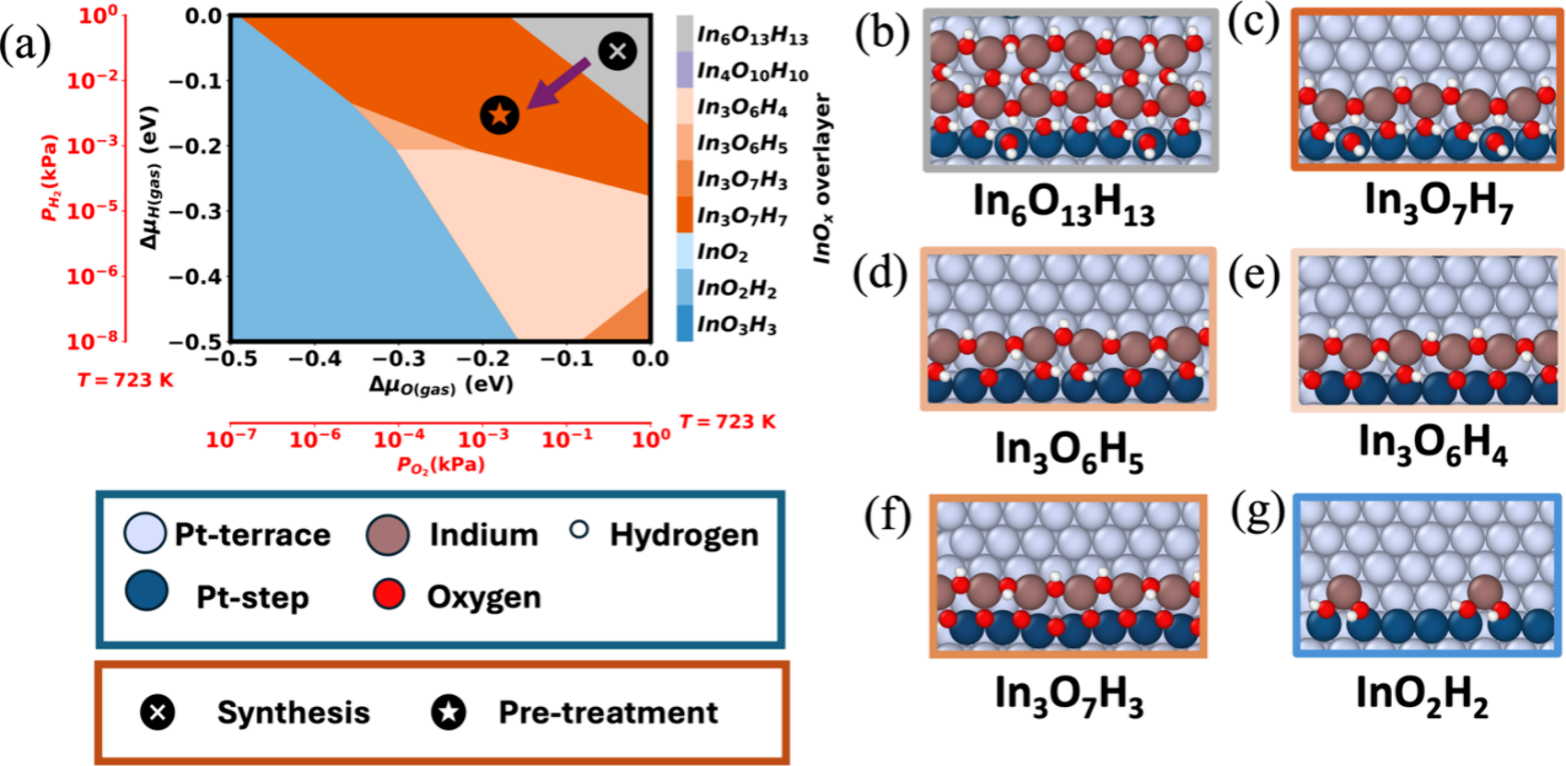
(a) Structural phase diagram of stable InO_*x*_H_*y*_ structures on Pt(322) at different chemical potentials of oxygen and hydrogen. (b–g) Stable structures as depicted in the phase diagram.

During synthesis, indium oxide is overcoated on Pt nanoparticles (NPs) using atomic layer deposition (ALD) at 150 °C.^7^ Given the ALD process involves an oxidant and hydrogen-containing species that form surface hydroxyl groups,^42^ stable InO_*x*_H_*y*_ structures under these conditions can be predicted using chemical potentials of O_2_ and H_2_ corresponding to partial pressures at and above the steady-state conditions of 5 and 1.32 kPa, respectively. Analyzing the phase diagram in Figure 2a with Δμ_i(gas)_ equal to zero reveals that In_6_O_13_H_13_ represents the most thermodynamically stable structure on the Pt surface in the presence of a bulk In_2_O_3_ reservoir. Sensitivity analysis at elevated partial pressures of the O_2_ and H_2_ gases above the aforementioned conditions confirms that In_6_O_13_H_13_ remains stable under excess O_2_ and H_2_ environments (Supporting Information, 3.1). As shown in Figure 2b, this structure predicts that the Pt surface is entirely covered by the InO_*x*_H_*y*_ overlayer under synthesis conditions. This is in line with the catalyst structure reported by Yan *et al.*,^7^ wherein the Pt surface is fully covered with indium oxide after synthesis using ALD and before the pretreatment. Following synthesis, the overcoated catalyst was subjected to pretreatment in a reactor where the temperature was increased to 450 °C under a nitrogen atmosphere.^7^ To understand the structure evolution under these conditions, the phase diagram is analyzed at decreasing chemical potentials of O_2_ and H_2_ gases to mimic the nitrogen environment with residual gas-phase amounts (O_2_ and H_2_) during pretreatment. Under these conditions, it is revealed that the In_6_O_13_H_13_ structure could transform to a set of overlayer structures with In coordination of three at ∼0.39 ML InO_*x*_H_*y*_ coverage as depicted in Figure 2c–f. These structures include In_3_O_7_H_7_ (Figure 2c) with the maximum number of OH* at the Pt-step sites, followed by In_3_O_6_H_5_, In_3_O_6_H_4_, and In_3_O_7_H_3_ (Figure 2d–f), which are stable at relatively low H-chemical potentials. All of these structures shown in Figure 2c–f consist of the InO_*x*_H_*y*_ phase interacting with every Pt-step site through bridging O* or OH* species. A further decrease in the chemical potential results in the formation of the InO_2_H_2_ structure with a low coverage of ∼0.1 ML, shifting the In coordination to two, as shown in Figure 2g. However, such low coverages of InO_*x*_H_*y*_ appear at extremely low O_2_ and H_2_ partial pressures and are unlikely to form compared to the ∼0.39 ML InO_*x*_H_*y*_ phases. This indicates that the plausible overlayer structures that result from pretreatment are in the form of ∼0.39 ML three-coordinated InO_*x*_H_*y*_ phases on Pt(322). Based on our phase diagram analysis, the structures in Figure 2b–f thus reveal a structural rearrangement of synthesized InO_*x*_ overcoat on Pt to preferentially decorate around the Pt-step sites during pretreatment.

We next compare the predicted structural evolution of the catalyst during pretreatment with experimental results. Experimentally, pretreatment resulted in the formation of a porous structure uncovering around half of the Pt active sites, as evidenced by an increase in the intensity of the CO diffuse reflectance infrared Fourier transform spectroscopy (DRIFTS) feature associated with Pt well-coordinated sites in the Pt-InO_*x*_H_*y*_ catalyst with temperature.^7^ Based on the comprehensive phase diagram analysis and the experimental observations by Yan *et al.*,^7^ we predict the three-coordinated ∼0.39 ML InO_*x*_H_*y*_ phase to be the dominant overlayer structure resulting from pretreatment, as it approximately consists of 50% of free Pt sites for the ODHP reaction (Supporting Information, 2.3). The arrow in Figure 2a illustrates the shift on the phase diagram from synthesis to pretreatment, depicting the corresponding structure change from In_6_O_13_H_13_ to ∼0.39 ML InO_*x*_H_*y*_ phase on the Pt(322) surface. In line with this, the CO-DRIFTS spectra also showed a reduced intensity of features associated with the under-coordinated Pt sites compared to uncoated Pt NPs.^7^ Further, the surface diagrams at different pretreatment temperatures show that the area of the contour region representing In_6_O_13_H_13_ reduces with an increase in temperature, indicating a decrease in thermodynamic stability of In_6_O_13_H_13_ across a specific range of H_2_ and O_2_ chemical potentials (Supporting Information, 3.2). Therefore, *the phase diagram analysis reveals that the experimentally observed increase in Pt sites during pretreatment could result from the destabilization of the InO*_*x*_ *H*_*y*_ *structure, specifically on the Pt-terrace sites, leading to their increased availability for PDH reaction.* This reconstruction of InO_*x*_H_*y*_, uncovering the Pt-terrace sites while preferentially decorating the Pt-step sites, could be a key factor in the pore formation observed experimentally in the Pt-InO_*x*_H_*y*_ catalyst during pretreatment.^7^ Our phase diagram further predicts that in addition to temperature, a precise control of O_2_ and H_2_ environments in the reactor during pretreatment is essential to drive this thermodynamically favorable site-selective reconstruction of InO_*x*_ on the Pt surface. This analysis showcases the potential effectiveness of a combined analysis of the atomistic phase diagram with experimental characterization to elucidate the active site structure of complex metal–metal oxide interfaces. To further predict the dominant phase under reaction conditions, we assume that the H_2_ partial pressure is high enough (>10^–4^ kPa H_2_) to maintain the three-coordinated In_3_O_7_H_7_ structure on Pt(322) as the active catalytic phase during the ODHP reaction. Using this structure, we evaluate the thermodynamics and kinetics of the ODHP reaction networks to understand the role of the InO_*x*_H_*y*_ overlayer in driving the catalytic reaction.

In summary, this computational investigation reports the ALD-deposited structure of InO_*x*_H_*y*_ on the Pt surface at high temperatures, offering insights into the relationship between the synthesis conditions and the resulting surface morphology. To the best of our knowledge, this represents the first study reporting the atomic structure of InO_*x*_H_*y*_ on a defective Pt surface and demonstrates qualitative agreement with the experiments. While this work employs thermodynamic analysis for structure prediction, it is crucial to understand that for other systems, kinetic effects can also play an important role. Nevertheless, the current approach provides an initial framework for elucidating the dynamic behavior of ALD-deposited metal oxides on a metal through atomic-level insights into metal–metal oxide interfaces.

### Oxygen Activation on the Pt-InO_*x*_ Catalyst

The reaction mechanistic analysis of the ODHP reaction on the Pt-InO_*x*_H_*y*_ catalyst with the identified stable In_3_O_7_H_7_ phase is performed in two stages. First, the SHC mechanism is analyzed on the Pt-InO_*x*_H_*y*_ catalyst, followed by the analysis of the PDH mechanism. Evaluating oxygen activation on the Pt-InO_*x*_H_*y*_ structure is crucial for understanding the role of InO_*x*_H_*y*_ in coupling the PDH reaction with SHC to maintain propylene selectivity and minimize overoxidation of PDH reaction intermediates.^23^ The activation of oxygen on Pt(322)@*In_3_O_7_H_7_* is assessed based on the binding energy trends of oxygen adsorption and water formation on the catalyst, as shown in Figure 3. The SurfGraph algorithm is then used to systematically place the oxygenated reaction intermediates (O* and OH*) at all unique possible sites on the Pt(322)@*In_3_O_7_H_7_* surface,^37^ and the binding energy is computed at 723 K using oxygen at 5 kPa and hydrogen at 1.32 kPa, respectively (Supporting Information, 1.2). Based on Figure 3, the oxygen gas molecule is dissociatively activated on the Pt-InO_*x*_H_*y*_ surface with a kinetic barrier of +0.53 eV to form O*, assuming O_2_* adsorption from O_2_(gas) has no significant thermodynamic barrier on the Pt surface (Supporting Information, 4.1). The most stable configuration for O* adsorption is at a 3-fold site formed by a Pt-step atom and two Pt-terrace atoms, as shown in inset b of Figure 3. The binding energy of O* adsorption is predicted to be −0.48 eV, indicating thermodynamically favorable oxygen adsorption. While step edge sites typically enhance the reactivity of oxygen activation,^43^ the presence of InO_*x*_H_*y*_ prevents potential overbinding of O* on under-coordinated Pt sites and facilitates the removal of O* as H_2_O­(g), as shown in the following analysis.

**Figure 3:**
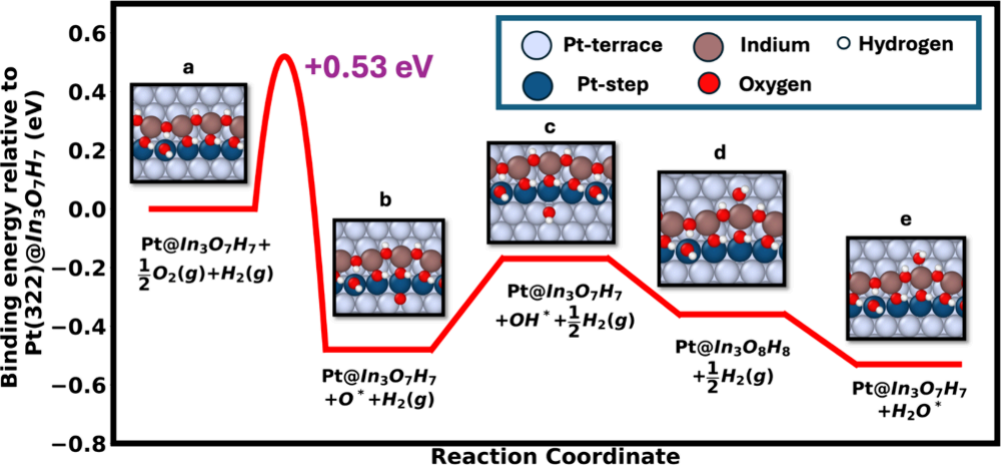
Binding energy trends to understand oxygen activation and the SHC mechanism on the Pt(322)@*In_3_O_7_H_7_* catalyst. Here, +0.53 eV represents the kinetic barrier for oxygen molecule dissociation. Insets (a)–(e) represent the stable structures of Pt-InO_*x*_H_*y*_ with their respective adsorbates mentioned in the inset text.

With H* available on the Pt surface from the dehydrogenation of hydrocarbon intermediates, O* combines with H* to form OH* at the Pt sites with a modest thermodynamic penalty of +0.3 eV (inset c of Figure 3) relative to O* on Pt(322)@*In_3_O_7_H_7_*. Further, the most stable site for OH* adsorption is identified as the bridging In and Pt-site forming In_3_O_8_H_8_, as shown in inset d of Figure 3. This is stable by −0.2 eV relative to OH* binding on Pt-terrace sites alone (inset c of Figure 3). Hence, our analysis predicts that OH* initially forms on Pt sites through combustion of H*, and InO_*x*_H_*y*_ would then consume OH* to form a stable hydroxylated surface. With OH* bridging Pt–In sites, the formation of water occurs with thermodynamic stability of −0.17 eV, relative to In_3_O_8_H_8_ (regenerating the In_3_O_7_H_7_ active site) as shown in insets d and e of Figure 3, and by −0.3 eV relative to the formation of water from OH* on the Pt-terrace site (Supporting Information, 4). Therefore, InO_*x*_H_*y*_ facilitates water formation with minimal thermodynamic and kinetic barriers, elucidating the mechanism for its high selectivity toward the SHC reaction.^44^ Comparison of water abstraction at different InO_*x*_H_*y*_ sites is further discussed in the Supporting Information, Section 4. Further, the current analysis reveals that InO_*x*_H_*y*_ plays an important role in reducing the amount of O* on the Pt surface, thus controlling overoxidation at the Pt sites. In summary, the mechanistic analysis of SHC reveals that it is a dual-site mechanism. O* activates on Pt sites, subsequently gets adsorbed at the InO_*x*_H_*y*_ active sites in the form of OH*, and the initial structure is restored when the additional OH* group leaves as water, completing the SHC catalytic cycle.

### Thermodynamics and Kinetics of ODHP on the Pt-InO_*x*_ Catalyst

The Pt-InO_*x*_H_*y*_ catalyst facilitates a facile SHC reaction with a low activation barrier for formation of O* and subsequent reduction to H_2_O. We now present a thermodynamic and kinetic analysis of the PDH reaction network on the Pt-InO_*x*_H_*y*_ catalyst. To understand the role of InO_*x*_H_*y*_ in selectively driving the PDH reaction, a comparative reaction mechanistic analysis is performed on three different catalyst models: terrace-like Pt(111), stepped Pt(322), and Pt-InO_*x*_H_*y*_ catalyst surfaces. For each system, stable adsorbate configurations are identified for key reaction intermediates, and the corresponding free energies and dehydrogenation barriers are estimated and reported in Figure 4.

**Figure 4:**
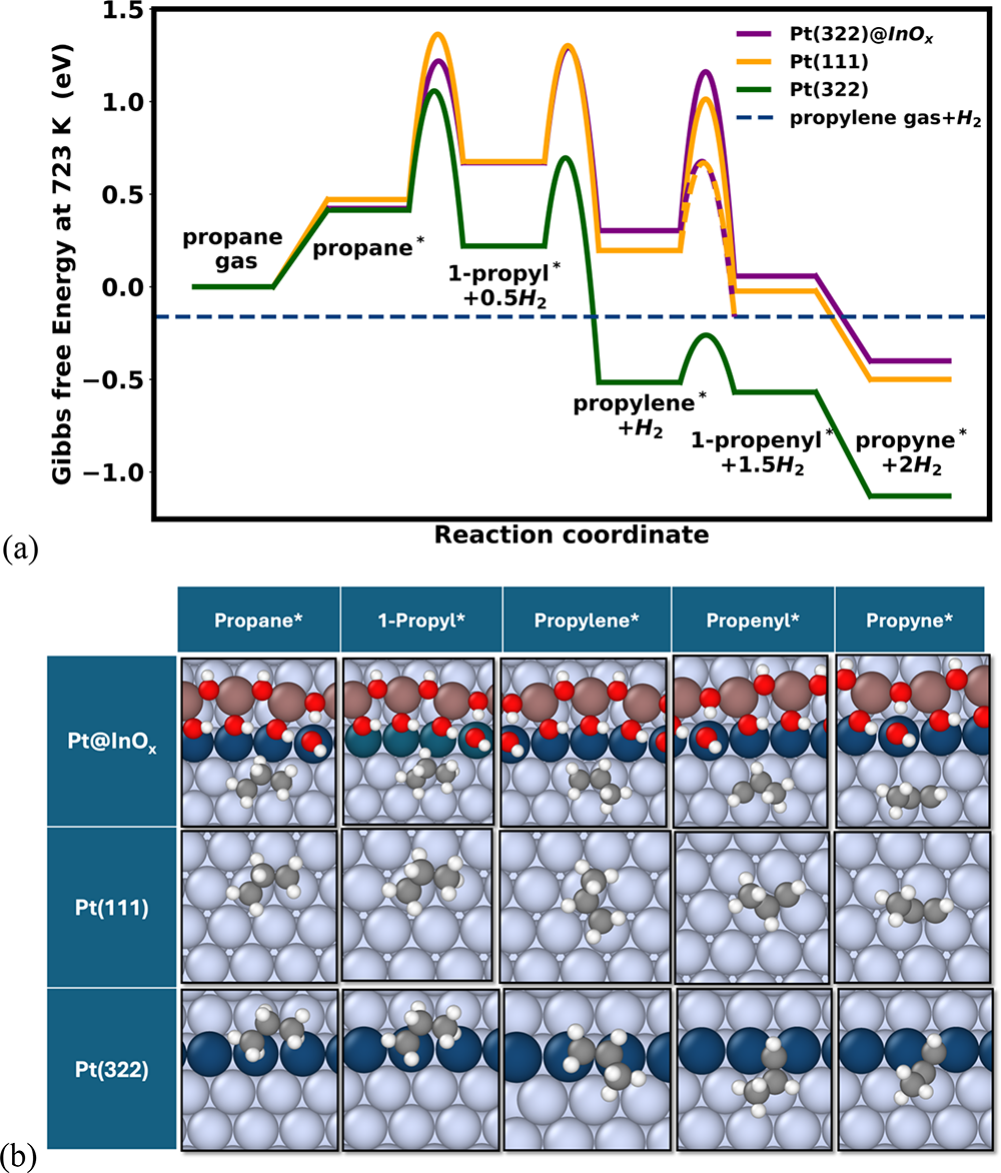
(a) Free energy diagram for propane dehydrogenation on Pt(322)@*In_3_O_7_H_7_*, Pt(111), and Pt(322) catalysts. (b) Thermodynamically stable configurations of key reaction intermediates of propane dehydrogenation on Pt(322)@*In_3_O_7_H_7_*, Pt(111), and Pt(322). Here, blue, light gray, brown, red, gray, and white correspond to Pt-step, Pt-terrace, indium, oxygen, carbon, and hydrogen atoms, respectively.

Stable configurations of the intermediates, 1-propyl*, propylene*, propenyl*, and propyne* are systematically identified by enumerating the adsorbate configurations on all unique Pt sites using the SurfGraph algorithm.^37^ Figure 4b presents the most stable adsorption configurations of these intermediates on the three surfaces. The preferred binding sites for 1-propyl*, propylene*, 1-propenyl*, and propyne* adsorption on all of the surfaces are consistently found to be on-top Pt, on-top Pt–Pt, bridge-top Pt–Pt, and bridge–bridge Pt–Pt sites, respectively. The adsorbates on the Pt(322) surface occupy the step sites due to their strong binding nature.^31^ In contrast, on the Pt-InO_*x*_H_*y*_ surface, the adsorbates occupy the Pt sites adjacent to the step sites as the step sites are saturated by the InO_*x*_H_*y*_ layer. Adsorption of all intermediates is weaker on Pt-InO_*x*_H_*y*_ and Pt(111) compared to Pt(322) due to the blockage of highly reactive step sites on the Pt-InO_*x*_H_*y*_ surface (Supporting Information, 7.1 ). Electronic structure analysis of Pt sites occupied by the InO_*x*_H_*y*_ layer shows that the d-band center shifts toward lower energy relative to the Fermi level, indicating decreased reactivity for adsorbate binding at those sites compared to Pt(322) (Supporting Information, 5).

A standard free energy diagram is then constructed and is shown in Figure 4a. It consists of the energetics of the dehydrogenation reaction, derived from the most stable adsorption sites of the PDH intermediates, and the reaction barriers for key elementary reaction steps, with propane gas as the reference. All of the intermediates on Pt(111) and Pt-InO_*x*_H_*y*_ show comparable thermodynamic free energies, suggesting similar adsorption thermodynamics on terrace sites. All the intermediates on Pt(322) exhibit more negative thermodynamic free energies due to their strong binding at the defect sites. The free energy of propylene* is a key feature governing the propylene gas desorption. On Pt-InO_*x*_ and Pt(111) catalysts, the thermodynamic formation of propylene gas from propylene* is downhill by 0.46 and 0.36 eV, suggesting favorable propylene desorption. However, on Pt(322), the propylene gas formation is uphill by 0.35 eV, predicting the requirement of additional energy for propylene desorption from the stepped surface. Similarly, the thermodynamic free energies of propenyl* on Pt-InO_*x*_H_*y*_ and Pt(111) catalysts are slightly higher than propylene gas formation by 0.22 and 0.13 eV, respectively, while the free energy of propenyl* formation on Pt(322) is lower than propylene gas formation energy by 0.4 eV, indicating higher propensity for deep dehydrogenation to propyne* over propylene desorption on Pt(322).

The kinetic analysis of different elementary steps in the PDH reaction also shows similar activation barriers for the Pt-InO_*x*_H_*y*_ and Pt(111) catalysts. The propane* activation or the first C–H bond breaking step in PDH is identified as the kinetically rate-controlling step on Pt-based catalysts.^31,45^ Comparison of the first dehydrogenation barriers on Pt-InO_*x*_H_*y*_, Pt(111), and Pt(322) in Figure 4a reveals that the propane* activation barrier is approximately equal on Pt(111) and Pt-InO_*x*_H_*y*_ (Pt-InO_*x*_H_*y*_ lower by 0.1 eV) due to similar active sites for adsorption, with Pt(322) exhibiting a lower barrier due to adsorption at reactive step sites. The dehydrogenation barriers of 1-propyl* and propylene* are also approximately equal on Pt(111) and Pt-InO_*x*_H_*y*_ due to similar potential energy barriers for C–H breaking of 1-propyl* (+0.78 eV on Pt-InO_*x*_H_*y*_ and +0.8 eV on Pt(111)) and propylene* (+0.89 eV on Pt-InO_*x*_H_*y*_ and +0.78 eV on Pt(111)). On Pt(322), lower dehydrogenation barriers are predicted as a result of the catalytic activity of step edge sites for C–H and C–C bond breaking.^46^ The activation energy difference between propylene dehydrogenation and propylene desorption serves as a selectivity descriptor on Pt metal and its alloys.^46^ Comparing the propylene* dehydrogenation barriers with the propylene* desorption barriers on Pt-InO_*x*_H_*y*_ and Pt(111) catalysts reveals that propylene* desorption is kinetically favorable on both catalysts, with approximately equal desorption barriers (Pt-InO_*x*_H_*y*_ lower by 0.1 eV) on Pt-InO_*x*_H_*y*_ and Pt(111). In contrast, Pt(322) has a higher desorption barrier, indicating low selectivity toward propylene gas formation (Supporting Information, 7.3 ). In summary, the presence of the InO_*x*_ layer modifies the adsorption thermodynamics and kinetics of PDH intermediates on Pt sites, enabling the exposed Pt sites to exhibit Pt(111)-like behavior and promoting favorable propylene formation and desorption.

Analyzing the role of indium oxide as an active site for PDH suggests that the C–H bond breaking barrier of 1-propyl* at the lattice oxygen sites is kinetically uphill by +2.5 eV, indicating unfavorable dehydrogenation on the InO_*x*_H_*y*_ layer (Supporting Information, 7.6 ). This is in line with the previous analysis where In_2_O_3_ has been proven to be a better catalyst for the SHC reaction due to its preferential selectivity for hydrogen combustion over hydrocarbon conversion.^44^ Therefore, this detailed analysis shows that the Pt-InO_*x*_H_*y*_ enables PDH reaction at the Pt-terrace sites with improved selectivity and SHC reaction at combined Pt-InO_*x*_H_*y*_ sites, controlling overoxidation of hydrocarbon intermediates at the Pt sites. The InO_*x*_H_*y*_ overlayer, therefore, plays a multifaceted role in the removal of H* formed during PDH while promoting a facile pathway for H_2_O formation through SHC and maintaining the desired coupling between PDH and SHC reactions.

## Discussion

Through our results, we have demonstrated that the probable active site structure of the Pt-InO_*x*_H_*y*_ catalyst is in the form of In_3_O_7_H_7_, which forms a continuous chain of a hydrogen-bonded network at the step sites. The proposed model predicts approximately ∼50% loss of majorly under-coordinated Pt sites, in line with the experimentally published results by Yan *et al*.^7^ Further, our mechanistic analysis shows that the OH* network plays a critical role in the ODHP reactions and protects the catalyst from deactivation. In this section, we aim to understand the role of InO_*x*_H_*y*_ in stabilizing the catalyst against deep dehydrogenation and coke deposition at the active site. Propyne* is a deep-dehydrogenated product of the PDH reaction, which has been proposed to be the starting point for the formation of coke and other undesired products since it preferentially cleaves the C–C bond over the C–H bond.^46^ The C–C scission of propyne* has been proposed to be the rate-controlling step for side reactions on the Pt catalyst.^47^ Therefore, understanding propyne* formation is crucial in assessing catalyst stability.

To specifically probe the effect of InO_*x*_H_*y*_ in stabilizing the catalyst against coking, we analyze the formation energies of propyne* on stable Pt-InO_*x*_H_*y*_ surfaces with varying O concentrations in the InO_*x*_H_*y*_ overlayer, as shown in Figure 5a. The O concentration in the InO_*x*_ overlayer directly correlates with the O-chemical potential as shown in the phase diagram analysis of stable InO_*x*_ structures at different O and H concentrations generated through the procedure described in Section 1 (Supporting Information, 6). At each O concentration, we selected the most stable InO_*x*_H_*y*_ configuration and analyzed the propyne* binding energy trends relative to the bare Pt(322) surface. The choice of the *x*-axis, which is the oxygen-to-indium ratio in Figure 5a, links the O_2_ gas concentration (O-chemical potential) in the reactor to the O/OH coverages in the Pt-InO_*x*_H_*y*_ structures. The images in Figure 5b show the corresponding propyne* configurations on Pt-InO_*x*_H_*y*_ structures at different O concentrations, estimated by enumerating propyne* at all unique Pt sites for each Pt-InO_*x*_H_*y*_ structure using SurfGraph algorithm.^37^ As shown in Figure 5a, with an increase in the O concentration in the InO_*x*_H_*y*_ overlayer, the formation energy for propyne* becomes unstable compared to the bare Pt(322) surface. This results from the decreasing availability of Pt-step sites with an increase in the O concentration in the InO_*x*_H_*y*_ overlayer, as shown in the images (1–6) of Figure 5b. At lower O concentration, propyne* occupies the step sites, as shown in Figure 5b images 1–2, with a binding energy of +0.25 eV relative to the Pt(322) surface. This slight decrease in binding energy is attributed to a decrease in the surface energy of the stepped Pt atoms due to the adjacent presence of the InO_*x*_ overlayer. A further increase in the O concentration results in an increase in the binding energy to +0.6–0.7 eV. This results from the complete blockage of the Pt-step sites with oxygenated groups of InO_*x*_ such as O*/OH* leading to the shift in propyne* binding to well-coordinated Pt-terrace sites as shown in Figure 5b images 3–6. Therefore, with step sites fully occupied by the chain of H-bonded network during the reaction, the most stable Pt(322)@*In_3_O_7_H_7_* stabilizes the catalyst by blocking the highly reactive Pt-step sites that are conducive to C–C bond breaking^31^ and coke formation.^48^ This suggests that the presence of InO_*x*_H_*y*_ with high O concentration controls deep dehydrogenation by weakening the binding of intermediates such as propyne* and suppresses catalyst deactivation.

**Figure 5:**
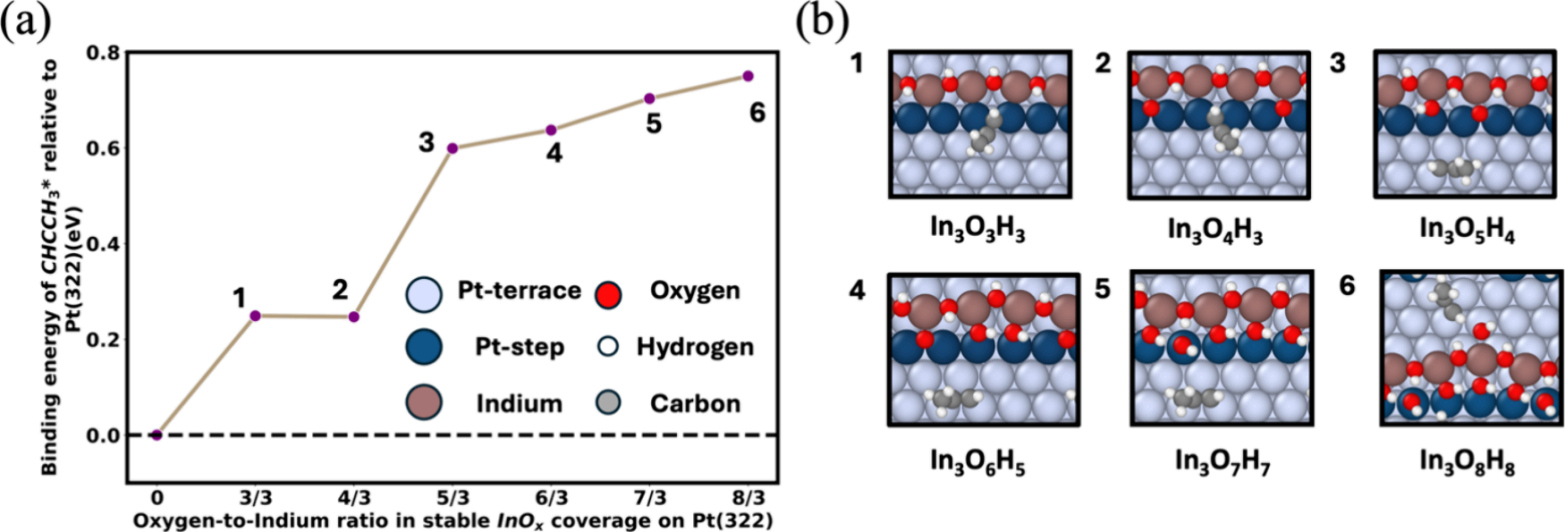
(a) Binding energy trends of the deep-dehydrogenated product, propyne* on Pt(322)@InO_*x*_ with an increase in the O/In ratio in stable InO_*x*_ coverage relative to Pt(322). (b) Images 1–6 represent the most stable surface structures of propyne* adsorbed on stable Pt@InO_*x*_ at each O concentration. With an increase in the O concentration, the binding energy increased, indicating a decrease in the stability of propyne*.

To further test this hypothesis, we computed the potential energies for other PDH intermediates on Pt(322)@In_3_O_3_H_3_ (Figure 5b image 1) and compared them with that on Pt(322)@*In_3_O_7_H_7_* (Figure 5b image 5) (Supporting Information, 7.7). These results showed a decrease in propylene* selectivity and an increase in selectivity toward deep-dehydrogenated intermediates for the case of Pt(322)@*In*_3_ *O*_3_ *H*_3_, in line with our hypothesis. This analysis solidifies the effect of the O/OH coverage in InO_*x*_H_*y*_ in stabilizing the catalyst. Hence, the sensitivity of the O concentration in the Pt-InO_*x*_H_*y*_ structure to the O-chemical potential could be used as an essential descriptor for assessing the stability and selectivity of M_1_ – M_2_ O_*x*_ H_*y*_ catalysts for ODHP. In summary, the analysis reveals that the performance of the M_1_ – M_2_ O_*x*_ H_*y*_ catalyst depends not just on the coverage and position of the oxide but also on the concentration of functional groups, such as O*/OH*, on the oxide surface. This alludes to the need for future research to integrate experimental and computational studies to identify the right synthesis conditions that lead to stable catalysts while understanding them at an atomic level, ultimately promoting physics-inspired optimal catalyst design.

## Conclusions

In this work, we developed a generalized theoretical workflow to unravel the atomic features of the tandem M_1_ – M_2_O_*x*_H_*y*_ catalyst that drive complex chemistries. We use this framework to analyze the overcoated Pt-InO_*x*_H_*y*_ catalyst, relevant for the selective and stable ODHP reaction. By exhaustively sampling the configurational space of the InO_*x*_H_*y*_ surface across different stoichiometries, the thermodynamically stable InO_*x*_H_*y*_ phases relevant to the synthesis, pretreatment, and reaction conditions are identified on the Pt(322) surface. Based on a phase diagram analysis, the experimentally observed pore formation during pretreatment of the catalyst is attributed to the destabilization of InO_*x*_H_*y*_ on Pt-terrace sites and preferential decoration around under-coordinated Pt-step sites. Our simulations, for the first time, provide atomic insights into the role of catalyst pretreatment under low H_2_- and O_2_-containing environments for the pore formation of ALD-deposited indium oxide on Pt metal at high temperatures. The key structural features that affect the stability of InO_*x*_H_*y*_ on the Pt(322) surface are identified as the coverage of In, the preferential interaction of In with Pt-step sites, and the number of hydroxyl groups forming hydrogen bonds at the Pt-step sites. These features can now serve as a starting point for future analysis to derive combined oxide and metal-based descriptors, thereby fundamentally understanding the oxide growth around different geometric features of a metallic surface. The identified stable Pt-InO_*x*_H_*y*_ atomic models are then used to explore the SHC reaction, which reveals a dual-site mechanism with thermodynamically feasible oxygen adsorption on Pt-terrace sites and subsequent adsorption of hydroxyl groups at the InO_*x*_H_*y*_ active sites to facilitate water formation without a significant thermodynamic barrier. Under these conditions, oxygen gas is shown to facilitate a continuous hydrogen bonding network at Pt-step sites, stabilizing the overcoat in the form of In_3_O_7_H_7_. Subsequent thermodynamic and kinetic analyses of the PDH reaction on Pt-InO_*x*_H_*y*_ in comparison to Pt(111) and Pt(322) reveal that the adsorption thermodynamics and kinetics of key PDH intermediates on Pt-InO_*x*_H_*y*_ are similar to those on Pt(111). The presence of the InO_*x*_H_*y*_ layer modifies the electronic properties of Pt-step sites, making them less reactive for hydrocarbon adsorption compared to terrace sites. Resembling the Pt(111) surface, Pt-InO_*x*_H_*y*_ increases the propylene* dehydrogenation barrier, thereby promoting propylene gas formation and enhancing catalyst selectivity. Further, by passivating Pt-step sites, which have been proposed to drive coke deposition, the InO_*x*_H_*y*_ overcoat controls catalyst deactivation and stabilizes the catalyst. Based on these insights, the extent of reducibility of the oxide is an important descriptor for selective ODHP. The ODHP selectivity is sensitive to reducibility as a highly reducible oxide could make the reaction susceptible to overoxidation, while an irreducible oxide could fail to selectively combust hydrogen. Furthermore, the sensitivity of the O concentration in the Pt-InO_*x*_H_*y*_ structure to the chemical potential of oxygen could be a crucial descriptor for evaluating the reactivity and stability of such catalysts. These insights, now for the first time, establish a direct structure–performance relationship for the Pt-InO_*x*_H_*y*_ tandem catalyst, explaining the experimental observations of pore formation and improved stable propylene yields. Importantly, this approach can be extended to other multifunctional catalysts involving metal–metal oxide frameworks and reaction environments, guiding the rational design of the next generation of overcoated catalysts with tailored selectivity and stability.

## Supplementary Material



## Data Availability

All DFT calculation data for this article are available in the Zenodo repository system. 10.5281/zenodo.17317025. Any queries related to materials or any other content of the work should be addressed to the corresponding author.

## Notes

Computation of gas-phase chemical potentials, structure exploration methodology and insights, phase diagram analysis at high/low partial pressures of O_2_ and H_2_ and different temperatures, oxygen activation at different Pt-InO_*x*_ sites, d-band center analysis, and thermodynamic and kinetic analysis of ODHP reaction (PDF)
