# Supplementary material for: Site-Selective Oxide Rearrangement in a Tandem Metal–Metal Oxide Catalyst Improves Selectivity in Oxidative Dehydrogenation of Propane
Source: J Am Chem Soc. 2025 Oct 28;147(45):41727–37. doi: 10.1021/jacs.5c13571 (PMC12616685; doi:10.1021/jacs.5c13571)
Supplement: Supplementary file 1 [file ja5c13571_si_001.pdf]

Supplementary Information: Site-Selective Oxide Rearrangement in Tandem Metal-Metal Oxide Catalyst Improves Selectivity in Oxidative Dehydrogenation of Propane

Snehitha Srirangam[1], Siddharth Deshpande[*,1]*

[1]Department of Chemical and Sustainability Engineering, University of Rochester, Rochester, NY 14627, USA

*Corresponding Author, Email: sdeshp10@ur.rochester.edu

Contents

1. Binding energy calculations……………………………………………………………..4

1.1 Computation of gas phase chemical potentials at p^0……………………………........4

1.2 Chemical potential of hydrogen gas during reaction…………………………..…....5

2. Structural insights from InO_xH_y overlayer on Pt(322) surface……………………........6

2.1 Methodology for structure exploration……………………………………..……....6

2.2 Effect of OH-groups………………………………………………………..…..…….8

2.3 Structure Evolution………………………………………………………………..…...9

2.4 InO_x coverage under reducing environment………………………………………..12

2.5 Exploration of partially hydroxylated structures with varying O and H……………13

3. Phase Diagram Analysis………………………………………………………………..14

3.1 Analysis at high partial pressures of Oxygen and Hydrogen…………………..…..14

3.2 Phase diagram at different temperatures……………………………………………...15

3.3 Phase diagram using multiple stable InO_xH_y structures…………………………...16

3.4 Sensitivity analysis at different number of layers of Pt-slab…………………………16

4. Oxygen Activation…………………………………………………………………………18

4.1 Oxygen Dissociation…………………………………………………………………..20

5. d-band center and strain analysis………………………………………………..……..20

6. Phase diagram at varying O-chemical potential……………………...…………………22

7. Thermodynamic and Kinetic Analysis………………………………………………….....23

7.1 Potential Energy for PDH reaction…………………………………….……...23

7.2 Free energy diagram under PDH conditions……………………………..……………24

7.3 Kinetic barriers for PDH reaction…………………………………………………..…..25

7.4 Second Pathway for ODHP on Pt-InO_x catalyst………………………………………26

7.5 C-O bond activation………………………………………………………………..……27

7.6 Role of InO_x for PDH reaction……………………………...……………...…..28

7.7 Potential energy diagram at different OH coverages…………………………………29

7.8 Pt lattice constant optimization…………………………………………………….…..30

7.9 Dipole and Spin Effects……………………………………………………………..…..31

References…………………………………………………………………...…………..32

1. **Binding energy calculations**

1.1 Computation of gas phase chemical potentials at p^0

The chemical potential of hydrogen gas (H_2) and liquid water (H_2O) can be calculated using the gibbs free energy equation as shown in Eqn. S1. Here E is the energy of single molecule from DFT, ZPE is the zero-point energy calculated using harmonic vibrational states, S is the entropy calculated using NIST data at 723 K. Table S1 lists the ZPE and S of H_2 and H_2O at 723 K. Table S2 lists the ZPE of adsorbates used in formation energy computation (Eqn. 1 in main manuscript). The chemical potential of oxygen gas is evaluated using free energy for $H_2O(g)$ formation in Oxygen Reduction Reaction(ORR) as shown in Eqn. 3 (main manuscript). The gibbs free energy for $H_2O(g)$ formation per proton at 723 K is calculated using enthalpy and entropy of water, hydrogen and oxygen gases using NIST data and is obtained as -1.12 eV.

$$G = E + ZPE - TS \qquad \text{(Eqn. S1)}$$

Molecule	ZPE (eV)	S (J/mol K) at 723 K
$H_2(g)$	0.28	156.55
$H_2O(g)$	0.57	220

Table S1: Zero point energies and entropies of H_2 and H_2O at 723 K

Molecule	ZPE (eV)
OH^*	0.36
H_2O^*	0.66
O^*	0.07

Table S2: Zero point energies adsorbates OH^*, H_2O^*, and O^* on Pt-InO_x surface.

1.2 Chemical Potential of hydrogen gas during reaction

Unlike oxygen, the partial pressure of hydrogen gas (P_{H_2}) in ODHP reaction cannot be directly ascertained due to its dynamic nature as it depends on the interplay of Propane Dehydrogenation (PDH) and Selective Hydrogen Combustion (SHC) reactions. Hydrogen is simultaneously generated via PDH and consumed in the SHC reaction during ODHP. Under specific reaction conditions, Yan *et al.* demonstrated that Pt-InO_xH_y exhibits high performance upto 14 hours of TOS, achieving propylene selectivity of 70%, propylene yield of 22%, and F_{tandem} of 40%.[1] The F_{tandem}, defined as (1 - H_2/C_3H_6) × 100%, serves as an indicator of the degree of coupling between the PDH and SHC reactions.[1] An F_{tandem} of 100% indicates that hydrogen acts as a limiting reactant and gets entirely consumed in the ODHP reaction, resulting in a near-zero partial pressure in the gas-phase, representing ideal kinetic coupling between PDH and SHC. However, the actual partial pressure of hydrogen gas during the reaction is governed by PDH and SHC reaction kinetics and other competing side reactions. Experimental findings indicate that the Pt-InO_xH_y catalyst maintains a nearly consistent degree of coupling and propylene selectivity.[1] Hence, the partial pressures of propylene ($P'_{C_3H_6}$) and hydrogen (P'_{H_2}) gases can be estimated by assuming steady-state conditions, wherein the concentrations of these gases remain invariant with time. Using $P_{C_3H_8}$ at 10 kPa, propylene yield at 22% and F_{tandem} of 40%, the partial pressure of propylene ($P'_{C_3H_6}$) and hydrogen gas (P'_{H_2}) can be evaluated as shown in Eqn. S2 and S3 respectively.

$$P'_{C_3H_6} = \text{yield}*P_{C_3H_8} = 0.22*10\text{ kPa} = 2.2\text{ kPa} \quad \text{(Eqn. S2)}$$

$$P'_{H_2} = P'_{C_3H_6}*(1-F_{tandem}) = 2.2*(1-0.4) = 1.32\text{ kPa} \quad \text{(Eqn. S3)}$$

As shown in Eqn. S3, P'_{H_2} is the partial pressure of hydrogen gas under steady-state assumption, during the reaction on the Pt-InO_xH_y catalyst. Although the propylene yields were reported

between 20-30% experimentally with change in experimental parameters,[1] we approximate P'_{H_2} as a reference partial pressure to understand the structure evolution in the surface phase diagram in Fig. 2a (main manuscript). Further, while side reactions such as hydrogenation and combustion may influence the actual amount of hydrogen gas during the reaction, P'_{H_2} provides a reasonable estimate for understanding the InO_xH_y structure and the reaction kinetics due to a uniform coupling between the tandem reactions.

2. Structural Insights from InO_xH_y overlayer on Pt(322) surface

2.1. Methodology for structure exploration

The initial distances between Pt-In, In-In, In-O, and O-H are set as 2.5 Å, 3.2-3.9 Å, 1.8-2.6 Å, and 0.9-1 Å, respectively. The coverage of InO_xH_y is estimated as the surface area of InO_xH_y on the Pt surface relative to bare Pt surface, calculated using an image recognition algorithm.[2] For the coverage of InO_xH_y on Pt, each In-In pair is bridged by one OH^* group, each terminal In is placed with two terminal OH^* groups. Two distinct configurations are considered varying the placement of OH^* groups. The bridging OH^* groups are directed towards the step edge with rest of the OH^* placed towards the terrace, and conversely, the bridging OH^* are directed towards the terrace, with the rest placed towards the step, maintaining a coordination of three oxygen atoms on each In. Exploration of cyclic InO_xH_y structures using the depth-first search algorithm revealed the preference for chain-like InO_xH_y structures over closed-ring configurations at the step sites as shown in further sections. Hence, the ring-type structures are converted to open chains preserving the preferred coordination of In during structure generation.

In the directional graph methodology, directional graphs are constructed using oxygen atoms as nodes and O-O edges within 3.2 Å. Configurations with the maximum number of cyclic edges,

representing maximized H-bonding, are selected. Hydrogen (H) atoms are then oriented along the normal of the O-O atoms, with the normal rotated by 50 degrees. If H-atom is within 2.3 Å to In or 1.7 Å to another H-atom, the rotation direction is reversed. Adequate separation is ensured between In-H and H-H to avoid bond formation between In and H-atom or within H-atoms. Using this algorithm, only the structures with maximized hydrogen bonding distribution are considered for the structure search. These structures are relaxed using DFT and at each coverage, the evolutionary algorithm[2] is applied choosing the top stable structures with a formation energy difference within 0.75 eV-1.5 eV with the most stable structure of the previous coverage.

As shown by a previous analysis, incorporating evolutionary algorithm reduces the configuration space by a factor of 95% to $O(10^2)$ oxide structures.[2] However, the presence of H^* in InO_xH_y structures increases the number of possible configurations on Pt(322) surface to ~15000 as shown in Fig. S1 due to (i) placement of OH^* groups toward and away from step sites (ii) conversion of closed ring-type to open chain-like InO_xH_y and (iii) orientation of H^* due to multiple possible hydrogen (H) bonding combinations at O-O pairs in the InO_xH_y structures. The combination of evolutionary algorithm with the directional graph methodology for choosing structures with maximized distribution of H-bonding reduces the total number of configurations to ~$O(10^2)$ as shown in main manuscript. (Fig. 1b).

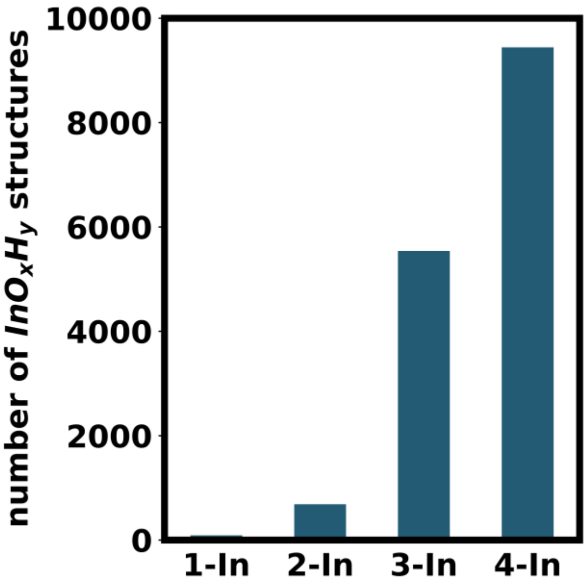

Figure S1: Number of identified possible unique InO_xH_y structures without applying the directional graph methodology for maximum distribution of H-bonding between O-O pairs.

2.2 Effect of OH-groups

The algorithmic framework is built assuming InO_x forms hydroxyl groups at each of its lattice oxygens. This is a valid assumption based on Fig. S2a and Fig. S2b where number of OH^* groups are increased at the bridging O^* atoms between each In-In pair and between each In-Pt-step site repectively. From Fig. S2a, OH^* groups at the bridging lattice oxygen sites between In-pairs enhances the binding strength of InO_x. The bridging OH^* groups between In and Pt-step sites also play an improtant role in maintaining the hydrogen-bonding network. As seen in Fig. S2b, the structure with OH^* groups at In-O-In is more stable compared to the structure with OH^* groups just at the In-Pt step site. However, the structure that has OH^* groups at every lattice oxygen site is the most stable structure with equal number of O and H atoms. The presence of these OH^* groups facilitates the formation of a chain of hydrogen bonds at the step sites. Further, as shown in Fig. S2c, exploration of structures varying the number of OH^* groups at the defect sites revealed that

increase in number of OH^* groups in H-bonding at the defect sites enhances the stability in the fully hydroxylated InO_xH_y structures. As the step sites are saturated, additional OH^* groups adsorb toward the Pt-terrace sites. However, these OH^* groups abstract in the form of water as discussed in next sections.

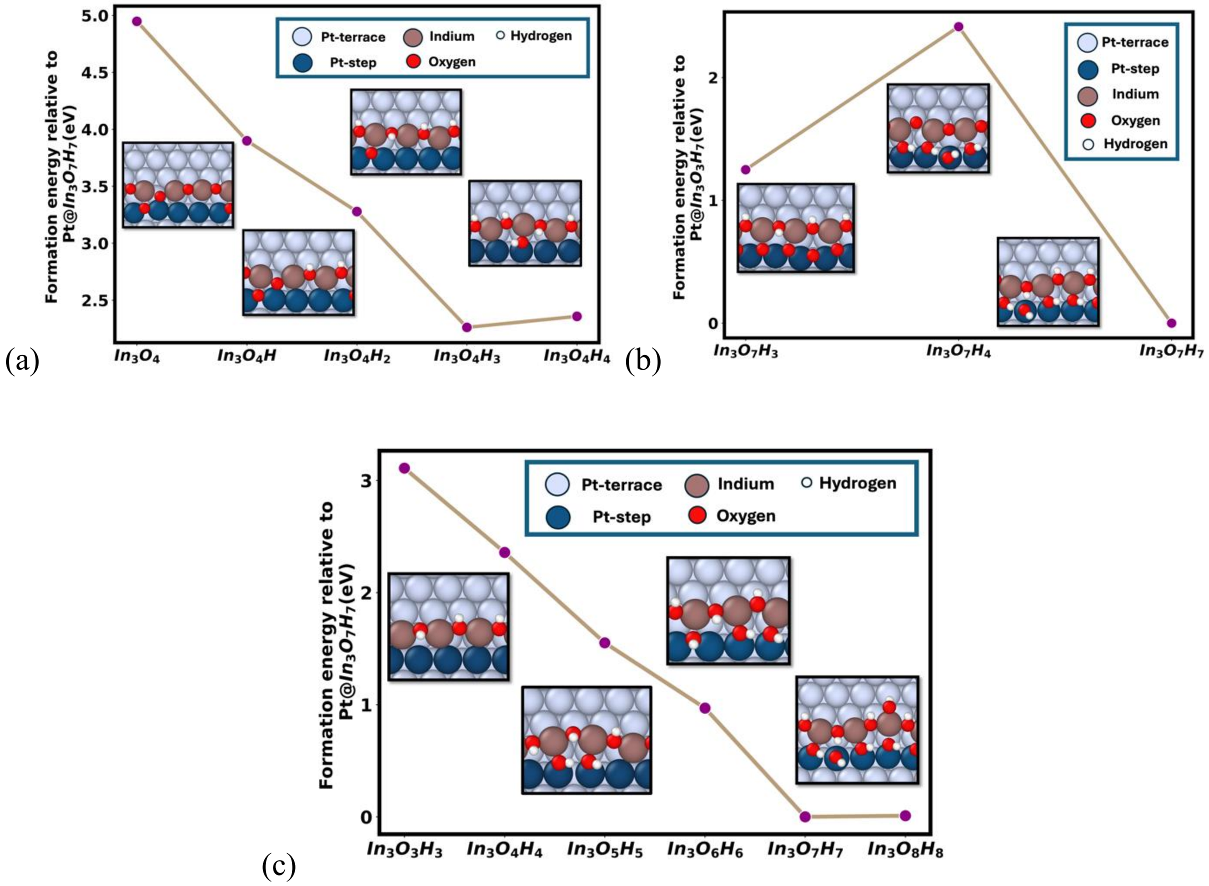

Figure S2: (a) Formation energy relative to $In_3O_7H_7$ with increase in hydroxyl groups on In_3O_4. (b) Formation energies relative to $In_3O_7H_7$ when there are bridging O* in the form of OH with O* at Pt-step sites, and step O* in the form of OH with bridging O* between In-pairs. The structure with all the O-atoms in the form of OH* is more stable. (c) Formation energy relative to $In_3O_7H_7$ with increase in OH* groups at the step sites. Once the step sites are saturated, the structure remains stabilized. All the energies are computed at 1.32 kPa of H_2 and 5 kPa of O_2.

2.3 Structure Evolution

The binding energy of stable structures of InO_xH_y at different coverages is computed with respect to Pt(322) utilizing the structure search methodology. Some of the top stable structures with

increase in number of InO_x units are shown in Fig. S3. At each coverage, in the most stable structure, In primarily occupies the Pt hollow sites adjacent to the step, while OH^* groups occupy the on-top sites of Pt-step. Each Indium atom is coordinated with at least one OH^* group bridging the In-Pt step site and atleast two OH^* groups bridging the In-In pairs. The structures that exhibit interactions with the Pt step sites are more stable compared to those without such interactions. From 1-In structures (0.13 ML) in Fig. S3, it is revealed that number of hydroxyl groups interacting with the step sites correlates with increased stability due to the hydrogen bonding between the OH^* groups. Based on 2-In (0.23 ML) and 3-In (0.39 ML) structures, in addition to number of OH^* groups at the step sites, interaction of In-atoms with the step sites is also found to be crucial in enhancing stability. Therefore, the main features that affect InO_x stability are the number of OH^* groups at the Pt-step sites, maximized hydrogen bonding and the interaction of In with Pt-step sites. In a unit cell containing four defective Pt-atoms, the metal oxide structure with three InO_x units covers the entire step, owing to larger atomic size of In compared to Pt. For 4-In (0.49 ML), 5-In (0.62 ML), and 6-In (0.73 ML) structures, once the step sites are fully saturated with InO_x units, the In-atoms preferentially form a cyclic structure ensuring each In-atom has atleast two bridging OH^* goups with other In-atoms. Overall, each In-atom has a coordination of three to four OH^* groups in the surface InO_x overlayer structure on Pt(322).

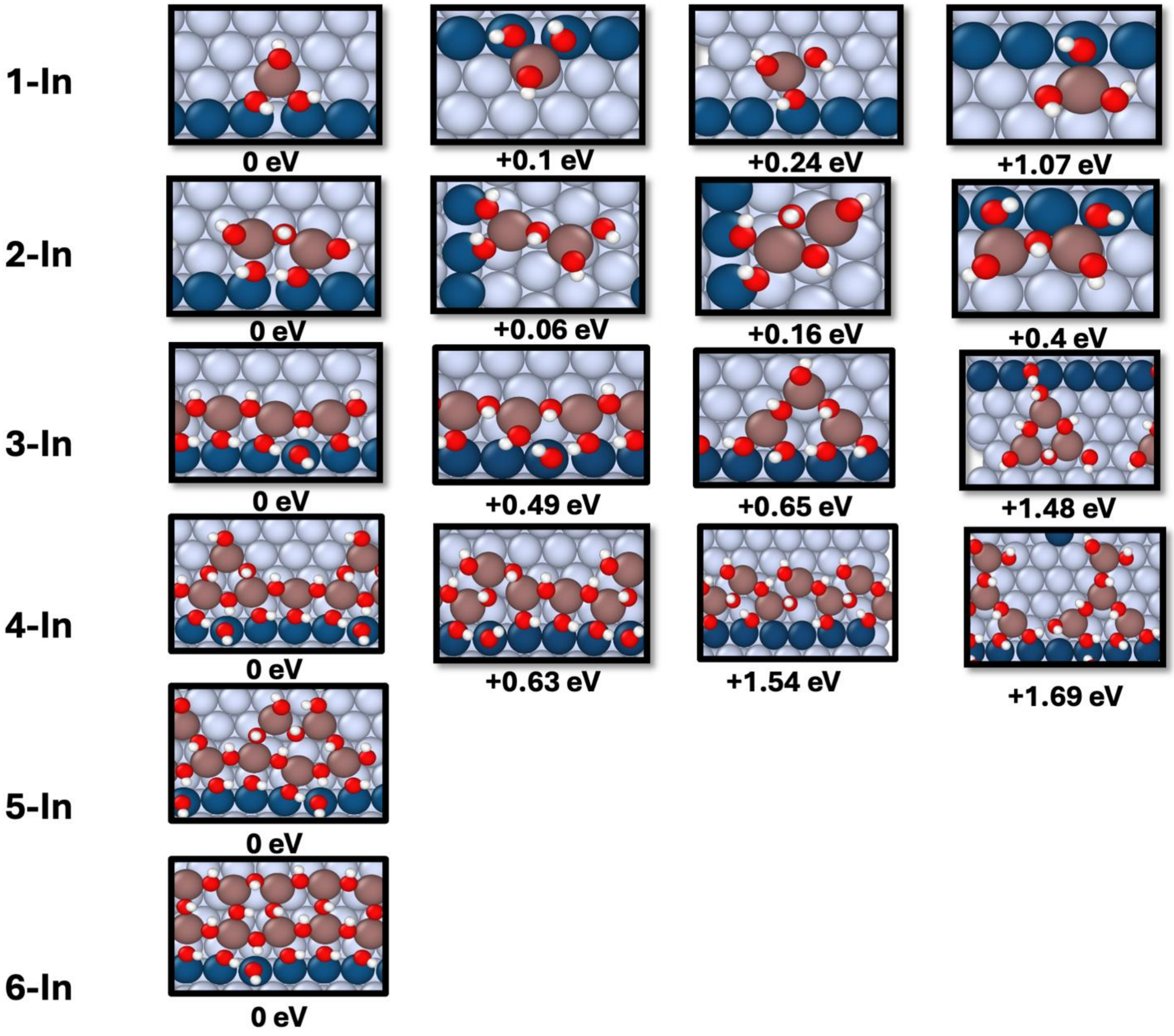

Figure S3: Top stable structures screened using the modified SurfGraph algorithm combined with evolutionary algorithm. The structures are placed in the order of increasing formation energy relative to most stable InO_x structures at each In coverage.

For the stable structure consisting of 3-InO_x units, eliminating the sites occupied by InO_xH_y as marked in blue in Fig. S4, the coverage of uncoated Pt sites is observed to be 0.483 ML. This shows at 3-In coverage, ~50% of the Pt sites are covered by InO_xH_y layer, exposing ~50% of uncoated Pt sites.

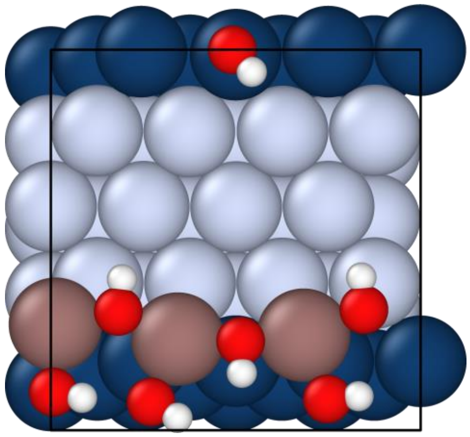

Figure S4: Unit cell consisting of uncoated Pt sites at 3-In coverage. Grey region represents uncoated Pt, while blue region represents coated Pt sites. Brown, red and white represent In, O, and H respectively.

2.4 InO_x coverage under reducing environment

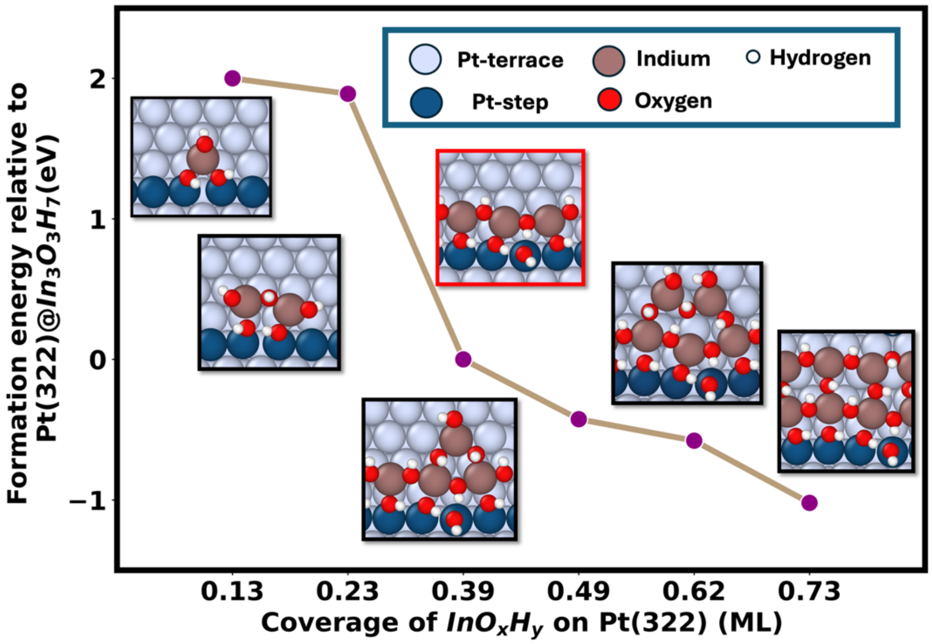

Figure S5: Coverage dependence of InO_xH_y structure on its formation energy relative to Pt(322)@$In_3O_7H_7$. At coverage of 0.73, the surface is entirely covered by InO_xH_y structure.These are subject to using μ'_{In} with respect to bulk In_2O_3, $\mu'_{H(gas)}$ with respect to the partial pressure of H_2 at the reactor output of 1.32 kPa and μ_O of 5 kPa.

2.5 Exploration of partially hydroxylated structures with varying O and H

Exploration of partially hydroxylated structures across a wider range of O and H chemical potentials requires screening of structures in the O(10^2) at each In coverage. To make the configuration space computationally tractable, we considered a set of assumptions as shown below.

1. Based on Fig. S2a and S2b, the O* bridging two In atoms is found to be stable in the form of OH* for a very large range of O and H-chemical potentials. Accordingly, we adopted the assumption that the O* bridging In-In remains in the form of OH*, while the oxygen species at the Pt step-In bridging sites can exist either as O* or OH*.
2. The bridge and top sites are considered for O* and OH* occupation on Pt-step respectively. Additionally, in cases where more than one OH* is present, the OH* are placed on adjacent Pt-step atoms to increase H-bonding between them. This is based on Fig. S2c, where it was observed that, the binding strength of the InO_x structure increases with the increase in number of OH* groups forming H-bonds at the Pt-step sites.
3. For an overlayer structure consisting of *n* O atoms, the number of H atoms is varied from *m* to *n* $(m<n)$. The rule is based on the finding that the number of H atoms cannot exceed the number of O atoms, assuming the SHC rate is fast on Pt-InO_x, and therefore, there are no isolated H* species. Here, *m* represents the number of H-atoms present in the form of OH* bridging In-In sites.

Based on the above framework, and using InO_x structure at 0.39 ML, $In_3O_yH_z$ structures were generated where $y = n$ and z ranges from 3 to n and stable configurations were identified for each combination of y and z. Fig. S6 shows the most stable $In_3O_yH_z$ structures at each y represented as the Oxygen-to-Indium ratio in the InO_x structures (insets a-e) and their respective formation energies relative to Pt@$In_3O_7H_7$ (~0.39 ML phase). One key feature that emerges from these

structures is that the structure that possesses maximum hydrogen bonding and interaction with maximum number of Pt-step sites is the most stable structure across different O- and H-concentrations under experimental conditions. An important point to note is that the stoichiometric ratio of $In_xO_yH_z$ obtained is subject to Pt(322) slab and can change for other slabs.

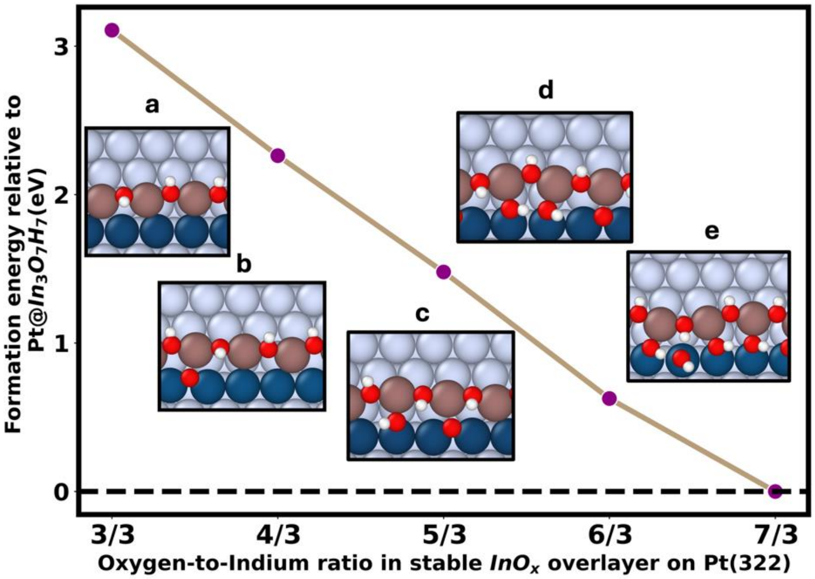

Figure S6: Formation energy relative to $In_3O_7H_7$ with increase in O/In ratio in the InO_x overlayer on Pt(322) surface. All the energies are computed at 1.32 kPa of H_2 and 5 kPa of O_2. Insets a-e represent the most stable InO_x structure at each O-concentration in InO_x represented as oxygen-to-Indium ratio.

3. Phase Diagram Analysis

3.1 Analysis at high partial pressures of oxygen and hydrogen

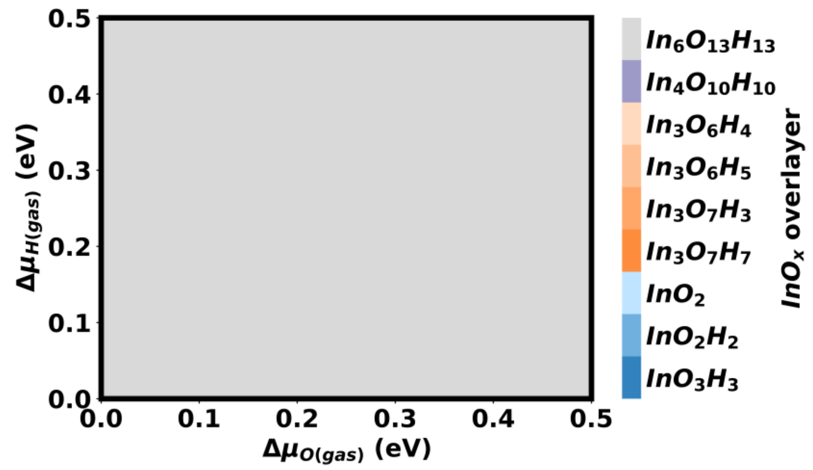

Figure S7: Phase diagram at partial pressures above the reference chemical potentials corresponding to partial pressures of 5 kPa and 1.32 kPa for O_2 and H_2 respectively. $In_6O_{13}H_{13}$ remains stable at partial pressures at and above the reference chemical potential under excess O_2- and H_2- environments.

3.2 Phase diagram at different temperatures

The synthesis of the Pt-InO_x catalyst is performed at 150^0C followed by pre-treament under nitrogen atmosphere. During the pre-treatment of the as-synthesized Pt-In_2O_3 catalyst, the catalyst is placed in a reactor and is heated until the temperature reaches 450^0C (723 K) under nitrogen atmosphere. Analysis of the phase diagrams at 388 K, 550 K and 723 K is performed considering the respective chemical potentials of O_2 and H_2 gas at those temperatures and is presented in Fig. S8. Fig. S8a represents the phase diagram at 388 K and Fig. S8b represents the phase diagram at 550 K. On comparing the phase diagrams at 388 K, 550 K and 723 K (main manuscript Fig. 2a), the area of the contour region representing $In_6O_{13}H_{13}$ reduces with increase in temperature. This implies with increase in temperature, there is a shift in the contour plots indicating the pressure ranges accessible by $In_6O_{13}H_{13}$ reduce, while there is an increase in the pressure range at which ~0.39 ML InO_x phases are stable. This indicates at a given pressure, the InO_x structure reconstructs from $In_6O_{13}H_{13}$ to ~0.39 ML InO_x phases with increase in temperature, in line with the experimental observation that a pore-like structure is observed with increase in temperature.[1]

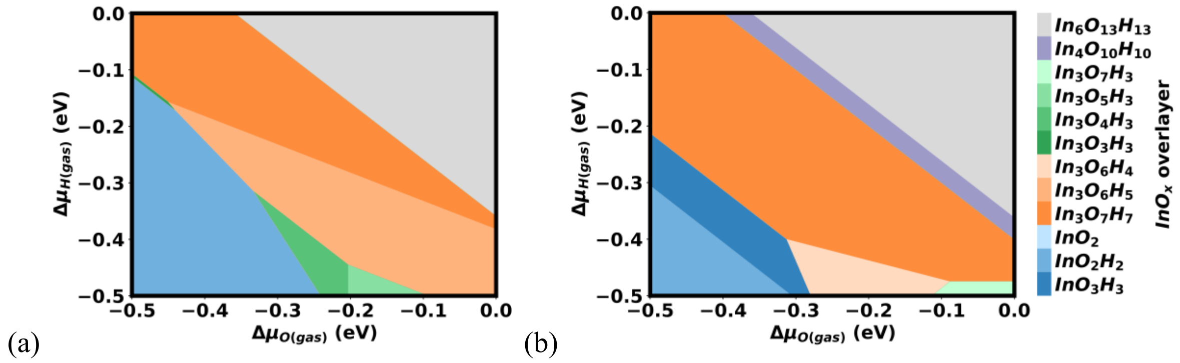

Figure S8: (a) Surface Phase Diagram during Pre-treatment at 388 K (b) Surface Phase Diagram during Pre-treatment at 550 K.

3.3 Phase diagram using multiple stable InO_xH_y structures

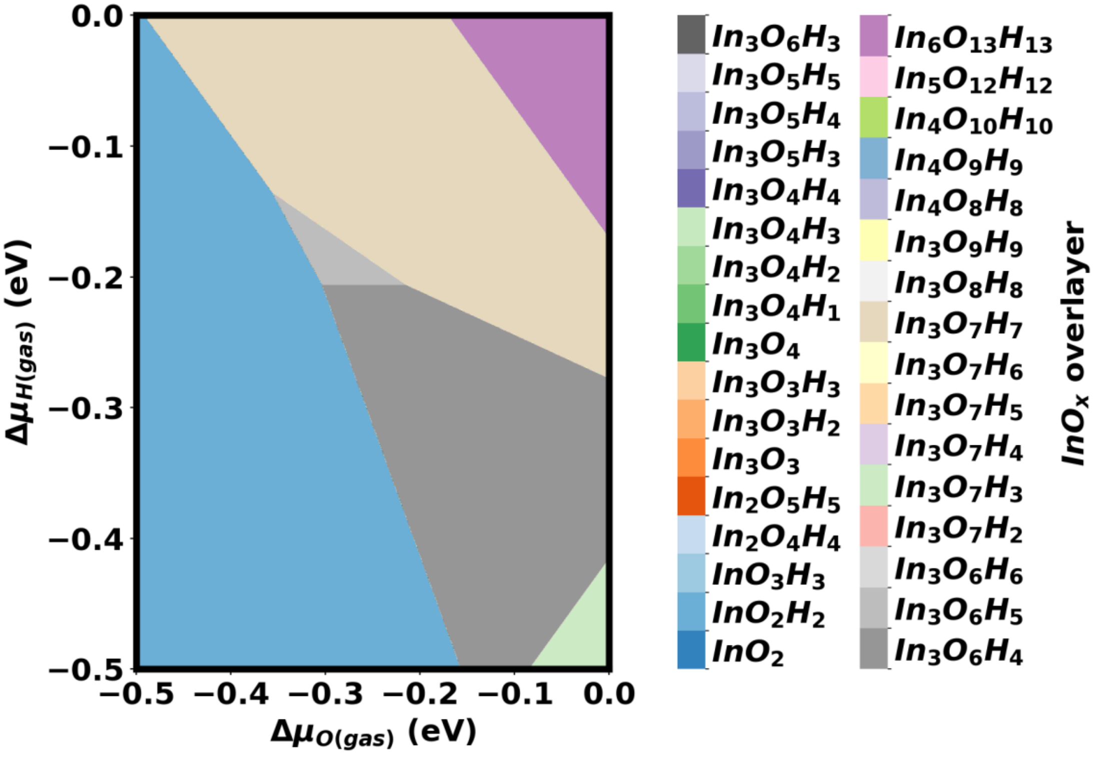

Figure S9: Phase Diagram constructed using multiple stable structures at 723 K. For brevity, only the dominant phases are presented in main manuscript. (Figure 2)

3.4 Sensitivity analysis at different number of layers of Pt-slab

We tested the effect of number of layers in the Pt-slab toward binding energy calculations and the phase diagram analysis. The Pt-slabs with different number of layers are presented in Fig. S10a. For each of the slab, the relative formation energies of dominant phases with respect to $In_3O_7H_7$ overlayer are computed and phase diagrams are constructed. With increase in number of layers from three to four, the average relative formation energy difference is obtained as 0.19 eV with a standard deviation of 0.14 eV. With increase in layers from four to five, the average energy difference is 0.08 eV with standard deviation of 0.06 eV, and an increase from five to six layers, the average energy difference is obtained as 0.07 eV with a standard deviation of 0.06 eV. However, the phase diagram presented in Fig. S10b shows that the conclusions about the dominant phases at different reaction conditions do not change with number of layers with the $In_6O_{13}H_{13}$ phase being stable under synthesis and the $In_3O_7H_7$ being the dominant overlayer after pre-treatment. While slab thickness may impact the mechanistic analysis, we proceed with three layer slab model since it is computationally less expensive for sampling large number of structures.

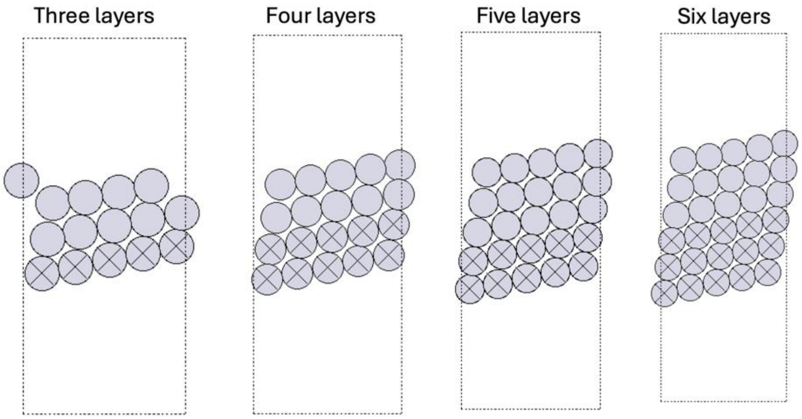

(a)

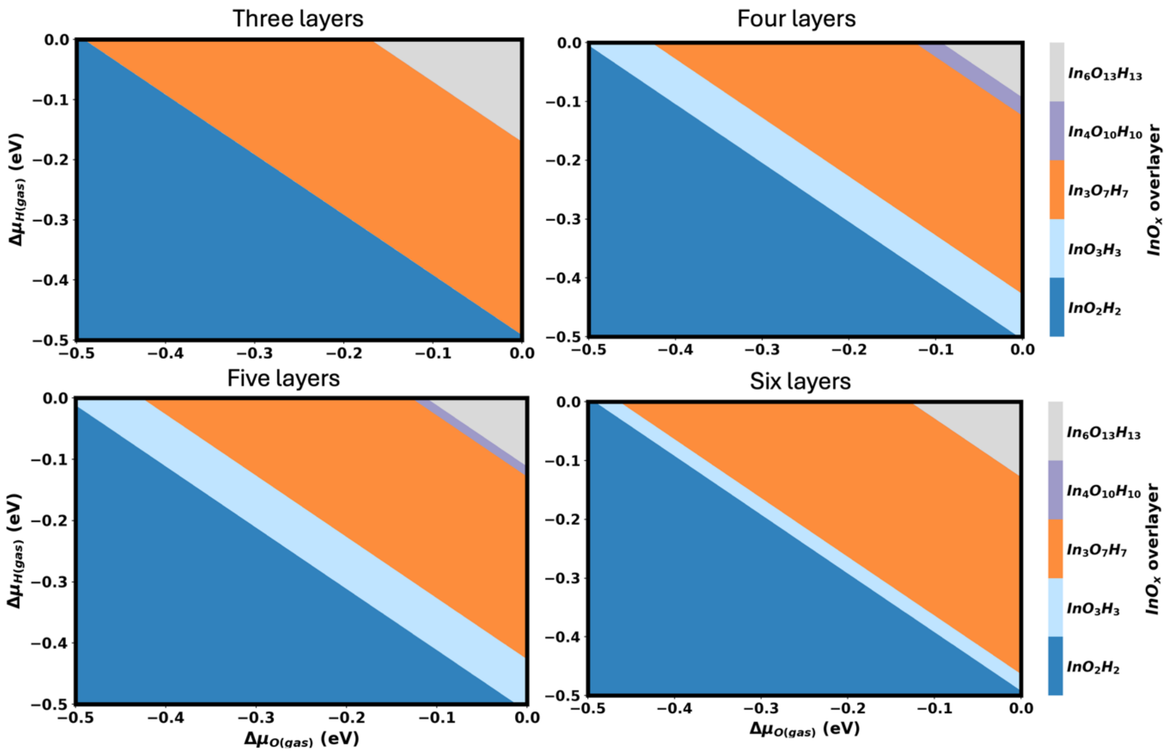

(b)

Figure S10: (a) Pt-slabs with different layers. (b) Phase diagram of InO_xH_y for different Pt layers

4. Oxygen Activation

The oxygen (O*) activated on the Pt-terrace sites on Pt-$In_3O_7H_7$ catalyst can undergo different pathways. As shown in Fig. S11a, the conversion of OH^* at Pt-terrace to H_2O^* has a small kinetic barrier around +0.3 eV for the formation of water. Therefore, O^* can facilitate the removal of H^* on the Pt-surface, driving the PDH reaction forward. The OH^* formed adsorbs at thermodynamically stable InO_xH_y active site and release as water with a kinetic barrier of +0.6 eV. These barriers can easily be overcome at a temperature of 723 K.

Comparing the water abstraction at different sites on InO_xH_y shows that water abstraction at the Pt-step site from step-OH^* that is not bound to In has a kinetic barrier of +0.6 eV as shown in Fig. S11b. However, the kinetic barrier for water abstraction at the OH^* bridging In and Pt-step atom

is observed to be +0.97 eV, indicating that water abstraction at the In-Pt-step sites is less stable compared to OH^* bridging In and Pt-terrace atoms (+0.6 eV). This suggests stronger interaction between In and Pt-step that influences the water formation at OH^* groups on the step sites, although this barrier can be overcome without an additional energy cost at 723 K. Another pathway for O^* activation is replenishing the InO_x sites with hydroxyl groups for driving selective hydrogen combustion (SHC) reaction. As OH^* desorbs as water at the Pt-step sites, these sites are replenished with OH^* group since Pt-step sites are thermodynamically stable sites for OH* adsorption.[3] Therefore, the primary role of oxygen is to facilitate the hydrogen-bonded network between In and Pt-step sites and maintain a stable hydroxylated InO_xH_y surface.

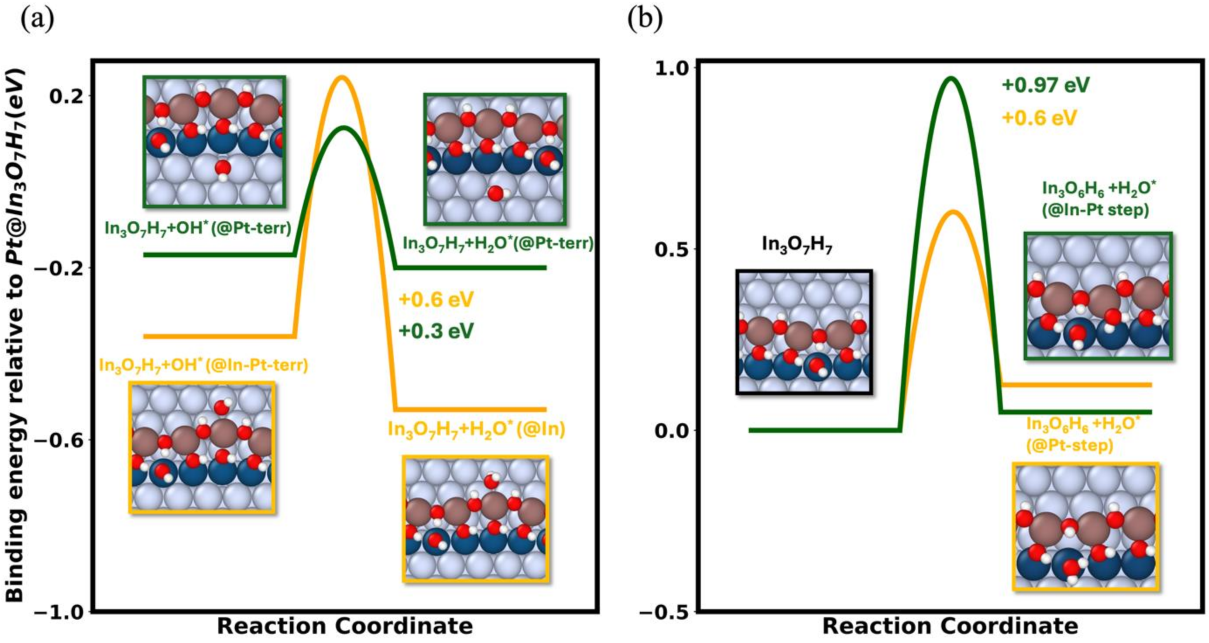

Figure S11: Binding energy trends for water formation at different sites on Pt and InO_xH_y active sites relative to Pt-$In_3O_7H_7$.

4.1 Oxygen Activation and Dissociation

Figure S12 shows the binding energy trends to form O^* from O_2 gas during SHC reaction. To understand the initial O_2* activation and dissociation on Pt, the SurfGraph algorithm is used to systematically place the di-oxygen intermediate at all unique possible sites on the Pt(322)@$In_3O_7H_7$ surface,[4] and the binding energy is computed at 723 K using oxygen at 5 kPa and hydrogen at 1.32 kPa, respectively. The O_2^* intermediate on Pt is assumed to be under quasi-equilibrium with the oxygen gas, including an entropy correction with 2-D ideal gas assumption.[5] Based on Fig. S12, the binding of O_2^* on Pt(322)@$In_3O_7H_7$ surface is thermodynamically stable by -0.51 eV at 723 K and the kinetic barrier for dissociation of O_2^* to two O* is estimated as +0.53 eV.

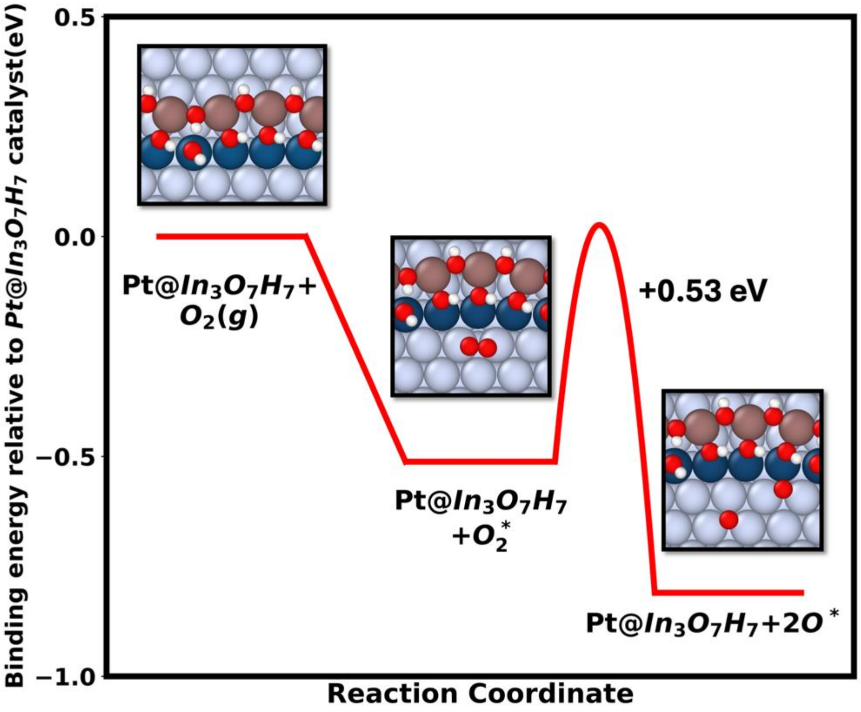

Figure S12: Binding energy trends of O_2* activation and dissociation on Pt@$In_3O_7H_7$ catalyst.

5. d-band center and strain analysis

To demonstrate the change in electronic structure due to the presence of InO_x overlayer, the atom projected density of states (DOS) is compared for overcoated Pt-InO_x and uncoated Pt(322) catalysts. Fig. S13a and S13b show the d-density of states for step and terrace sites respectively on both the surfaces. From Fig. S13a, along the step edge, there is a shift in the d-band center toward the bonding states, from -1.97 eV on Pt(322) to -2.4 eV on Pt(322)-$In_3O_7H_7$ surface. Presence of InO_x layer at the step sites leads to increase in the low energy states reflected in a shift of -0.43 eV to the left of the Fermi level. Further, in the InO_xH_y structure, Indium atoms occupy hollow sites on Pt spanning two terrace-like layers adjacent to the step. Computing the d-band center at a terrace layer occupied by InO_x shows a shift of d-band center by -0.2 eV from Fig. S13b. This shows a comparably smaller shift of d-band center at the terrace sites compared to step sites due to the presence of InO_x layer. Therefore, this analysis indicates strong interaction between Pt and InO_x sites at the Pt-sites that are covered by InO_x layer, specifically at the step sites with a higher d-band shift. Comparing the strain at the Pt-sites occupied by InO_x with Pt(322) showed no significant changes due to the presence of InO_x layer.

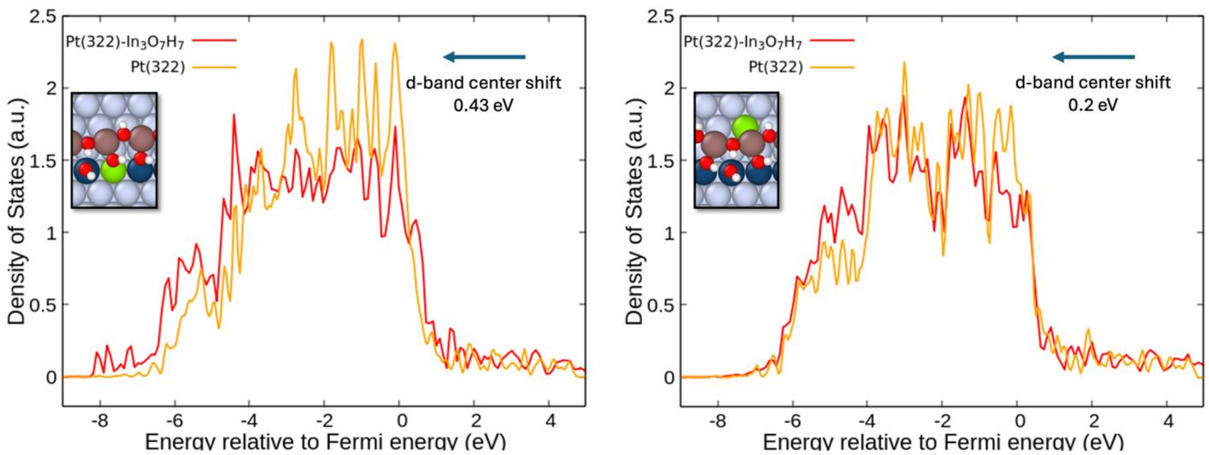

Figure S13: Atom projected d-orbital density of states for Pt(322)-$In_3O_7H_7$ and Pt(322) on (a) step site (b) terrace site. Inset shows the Pt(322)-$In_3O_7H_7$ structure where the density of states is computed for the green marked atom.

In the presence of adsorbates, the calculated d-band center at the adsorption sites of 1-propyl* on Pt(322)-$In_3O_7H_7$, Pt(111) and Pt(322) are -2.11, -2.09 and -2.08 eV respectively. This shows there is no significant shift in d-band center at the hydrocarbon adsorption sites due to the presence of InO_x layer at the Pt-step sites. A strain analysis also showed equal strain at all the surfaces at the hydrocarbon adsorption sites indicating no effect on strain of Pt-atoms due to InO_x layer. This shows InO_x has electronic effect on the occupied Pt-sites, increasing the binding energy of PDH intermediates at these sites. However, it has no geometric effects such as strain at any of the Pt-sites.

6. Phase Diagram Analysis of 3-In structures at varying O-chemical potential

Using the stable $In_3O_yH_z$ structures, we constructed phase diagram as a function of O-chemical potential (Fig. S14), to explain the conditions under which the partially hydroxylated structures are feasible. Our analysis reveals that the overlayer structure is sensitive to changes in O-chemical potential and this serves as a key tuning knob in the reactor as discussed below. As shown in Fig. S14a, with decrease in O-chemical potential, the phase diagram shows a shift in the oxide phase from $In_3O_7H_7$ (Fig. S14b) to $In_3O_6H_5$ (Fig. S14c), followed by a set of low OH coverage phases such as $In_3O_4H_3$ and $In_3O_3H_3$ (Fig. S14d and Fig. S14e) which predominantly have step sites unoccupied by OH groups. This is due to the formation of unhealed vacancies from the SHC reaction, and lower kinetic barriers for water formation at the OH groups bridging step Pt-In sites compared to OH bridging In-In sites (the kinetic barriers for water formation at these sites are +0.97 eV and +1.13 eV, respectively). These structures are identified as the most stable InO_x configurations at their respective O-concentrations from Fig. S6. Therefore, the O-chemical potential is found to be directly correlated with the O-concentration in the InO_x overlayer structure.

This shows the O_2 gas concentration (O-chemical potential) plays an important role in maintaining high/low OH coverages in the Pt(322)@InO_x structures. However, relatively low partial pressures ($<10^{-8}$ kPa O_2) are needed to thermodynamically stabilize Pt-InO_x states with unhealed vacancies as shown in Figure S14a.

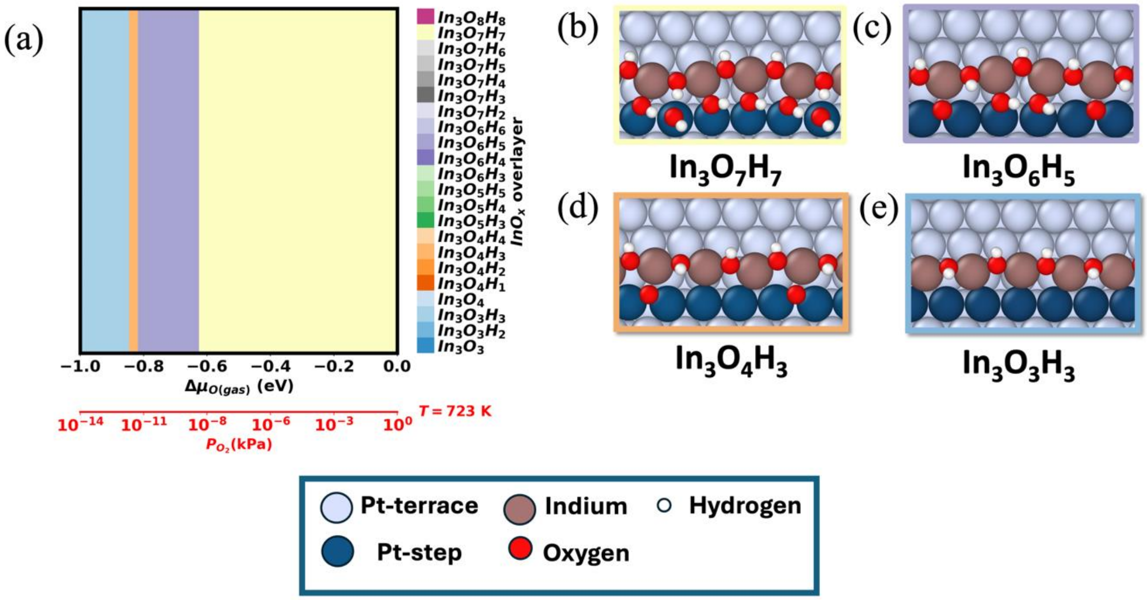

Figure S14: Structural Phase diagram of stable InO_xH_y structures with constant In-coverage of three at different chemical potentials of Oxygen at 1.32 kPa of Hydrogen. (b-d) Stable structures as depicted in the phase diagram.

7. Thermodynamic and Kinetic Analyses

7.1 Potential Energy Analysis for PDH reaction

Fig. S15 shows the potential energy diagram (without ZPE corrections) for PDH intermediates on Pt-InO_x catalyst in comparison to the uncoated Pt(111) and Pt(322) catalysts. It is revealed that the binding energy paradigm is similar on Pt(111) and Pt-InO_x catalysts. The trends observed in Pt(111) potential energy diagram align with previous findings.[6] These trends are in line with the conclusions derived from the free energy diagram in the main manuscript (Fig. 4).

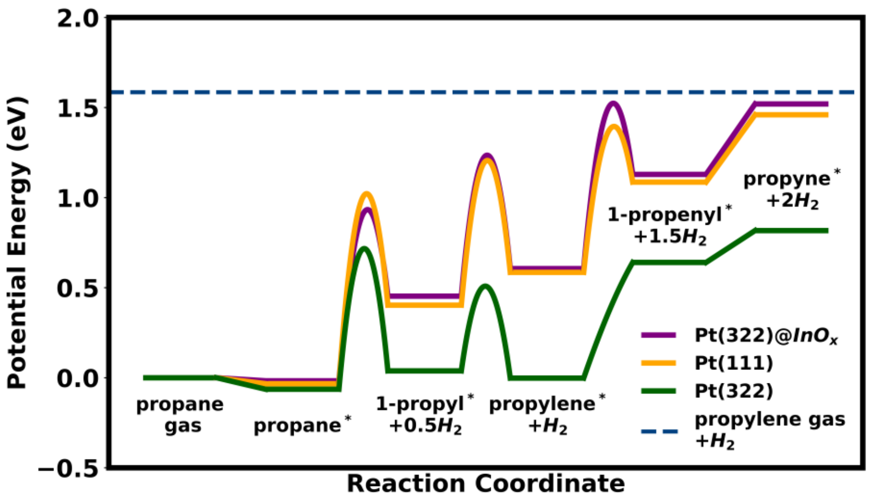

Figure S15: Potential Energy Diagram of dehydrogenation of key reaction intermediates for Propane Dehydrogenation on Pt(322)@$In_3O_7H_7$, Pt(111) and Pt(322) catalysts.

7.2 Free energy diagram under PDH conditions

Fig. S16 represents the free energy diagram under PDH conditions at a partial pressure of hydrogen at 1 bar. The gas phase energies of propane and propylene gas align with previous findings[6, 7] and thermodynamic free energies of the PDH intermediates on Pt(111) are in a similar range as previous findings.[6] The downshift in the thermodynamic free energies of PDH intermediates and propylene gas in the main manuscript (Fig. 4) is due to the consideration of an accurate partial pressure of hydrogen under ODHP reactor conditions at 1.32 kPa asusming steady-state, in contrast to a pressure of 1 bar under PDH reaction conditions.

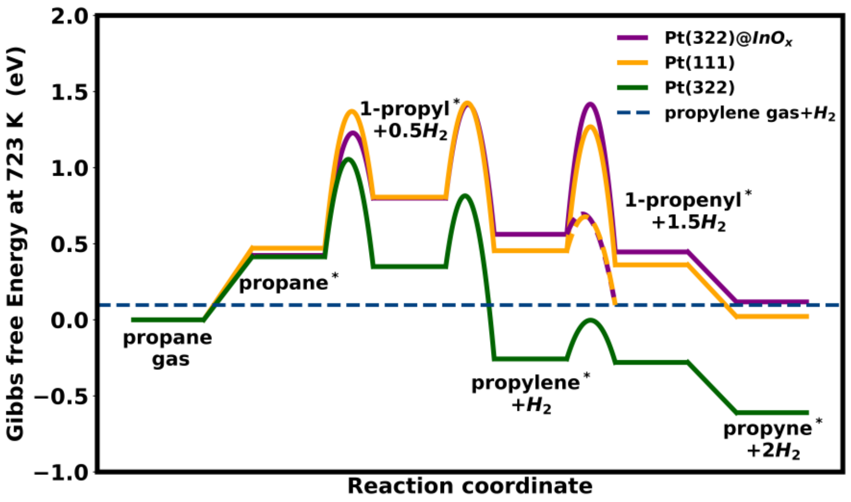

Figure S16: Free energy diagram under PDH conditions at 1bar H_2 chemical potential.

7.3 Kinetic barriers for PDH reaction

All the transition state energy barriers are calculated using CI-NEB calculations and the transition states are presented in Fig. S17. The desorption barriers of propylene* are calculated using 2-D ideal gas assumption. The enthalpy of the free energy barrier is taken as the desorption energy of propylene* to form propylene gas. The transition state entropy for Pt(111) and Pt@InO_x is assumed to be equal due to similar adsorption thermodynamics and kinetics on both the surfaces, as discussed in the main text. The entropy is calculated based on the formalism by Campbell *et al.*,[5] $S_{ads}(T) = 0.7S_{gas}(T) - 3.3R$ for terrace surfaces.[6] Using this approximation, the propylene* desorption barriers for Pt(111) and Pt-InO_x catalysts are obtained as +0.35 and +0.45 eV respectively. For stepped type Pt(322) surface, the entropy loses one translational degree of freedom and is assumed to be 1D ideal gas (1/3rd of that for a 3D ideal gas).[8] Based on this, the desorption energy barrier is observed to be +1.5 eV, which is much greater than propylene* dehydrogenation barrier of +0.25 eV on Pt(322). This is in line with previous studies,[9] indicating dominant presence of deep-dehydrogenated intermediates on the stepped Pt surfaces.

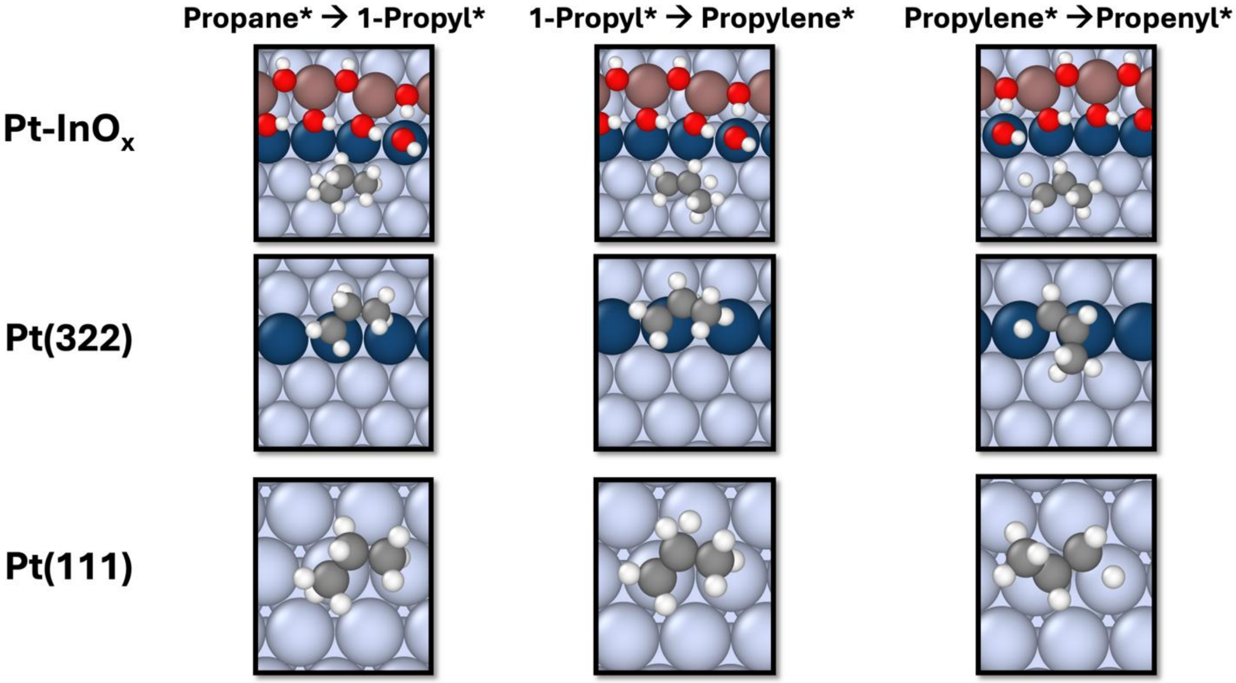

Figure S17: Transition state barriers for C-H activation of PDH intermediates propane*, 1-propyl* and propylene*.

7.4 Second Pathway for ODHP reaction on Pt-InO_x catalyst

In addition to the existing pathway described in the main manuscript (Fig.4), due to the presence of OH^* network at the Pt-step sites, Pt-InO_xH_y exhibits a second pathway for PDH involving direct transfer of H^* from hydrocarbon intermediates to form propylene gas and H_2O. Fig. S18 shows the kinetic barrier for direct transfer of H^* from 1-propyl* to OH^* at the InO_x sites. This step involves formation of propylene gas and H_2O through transfer of H^* from monodentate 1-propyl* to OH^* at Pt-step site without further dehydrogenation. This has a kinetic barrier of +0.86 eV, which is similar to C-H activation fo 1-propyl* to form propylene* on Pt terrace sites (+0.78 eV). The kinetic barrier for H_2O formation at OH^* bridging In-Pt step sites through this direct H^* transfer is uphill by +0.1 eV, showing no significant difference as shown in Fig. S18. This shows the ODHP reaction could also exhibit dual site mechanism similar to SHC at the Pt-InO_xH_y interface. Through this pathway

of direct hydrogen transfer, it minimizes deep dehydrogenation on Pt sites by eliminating the pathway towards formation of bidentate PDH intermediates.

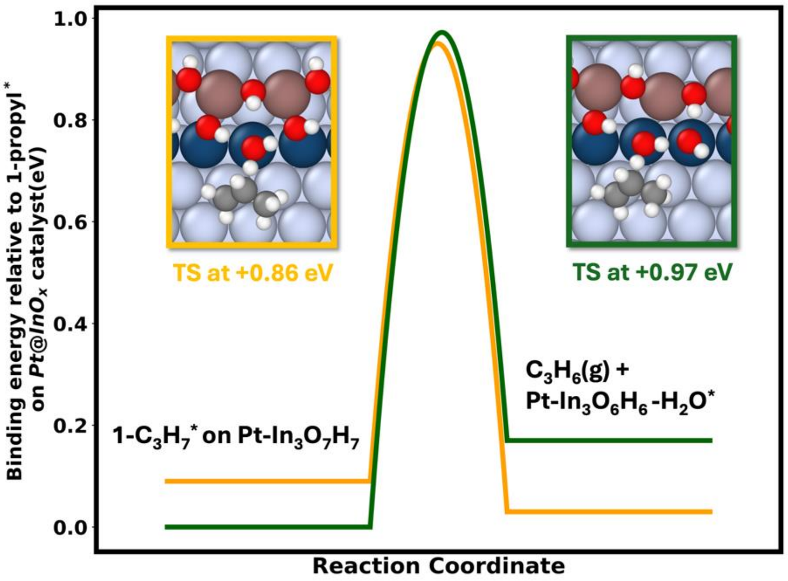

Figure S18: ODHP pathway from 1-propyl* to propylene gas through direct H* transfer to InO_x sites to form water.

7.5 C-O bond activation

In the presence of O*, there is no thermodynamic barrier for C-O activation in comparison to C-H activation of 1-propyl* on Pt-terrace sites as shown by the negative binding energy of oxygenated product in Fig. S19. However, CO-activation of 1-propyl* was found to have a higher kinetic barrier of +1.29 eV compared to the dehydrogenation of 1-propyl* at +0.87 eV from main manuscript (Fig. 4). This suggests that the kinetic barrier for C-O bond activation from 1-propyl* to form a combusted product is higher compared to the dehydrogenation barrier of 1-propyl* to form propylene* at the Pt-terrace sites. Further, in the presence of InO_xH_y layer on Pt-step sites, the pathway to oxidize 1-propyl* using step OH* is hindered due to strong hydrogen bonded network at the step sites as shown in next section.

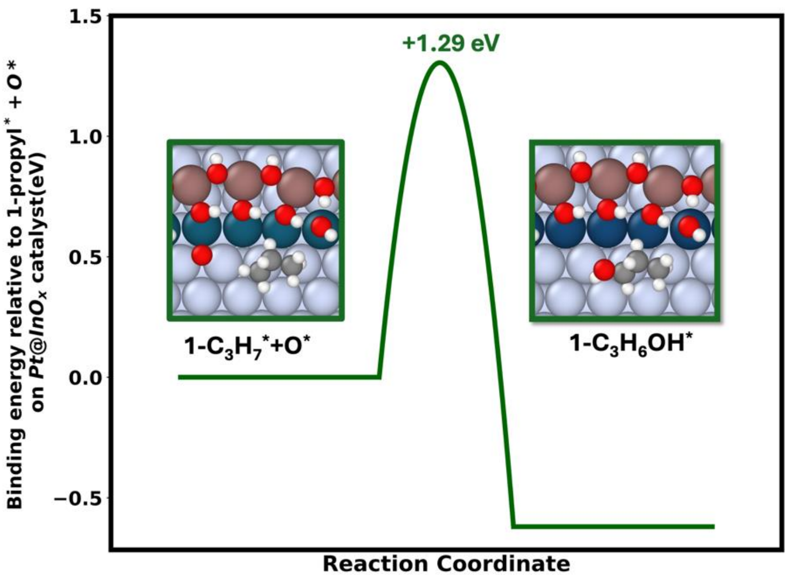

Figure S19: C-O bond activation in the presence of 1-propyl* on Pt@InO_xH_y catalyst.

7.6 Role of InO_x in PDH Reaction

Fig. S20 presents the role of InO_x in driving PDH reaction. The binding energy of adsorption of 1-propyl* at the lattice oxygen sites, as shown in inset in Fig. S20a, is as stable as its binding energy on the Pt-sites. The adsorption of 1-propyl* at the lattice oxygen site between In-pair is thermodynamically less stable by +0.17 eV compared to 1-propyl* on Pt-terrace sites. C-H activation of the 1-propyl* to form propylene gas at the lattice O* shows a thermodynamic barrier of +0.67 eV and a kinetic barrier at +2.5 eV in Fig. 20a. This shows InO_x is not favorable for 1-propyl* dehydrogenation in line with its high selectivity for SHC compared to PDH. Further, from Fig. S20b, adsorption of 1-propyl* at OH* on Pt-step can lead to formation of gas phase alochol. However, this has a high kinetic barrier of +2.0 eV due to 1-propyl* adsorption at less reactive step sites in the presence of InO_x layer and breakage of strong hydrogen bonded network, as shown in insets of Fig. S20b. This shows the primary role of InO_x in ODHP reaction is to selectively drive SHC over PDH.

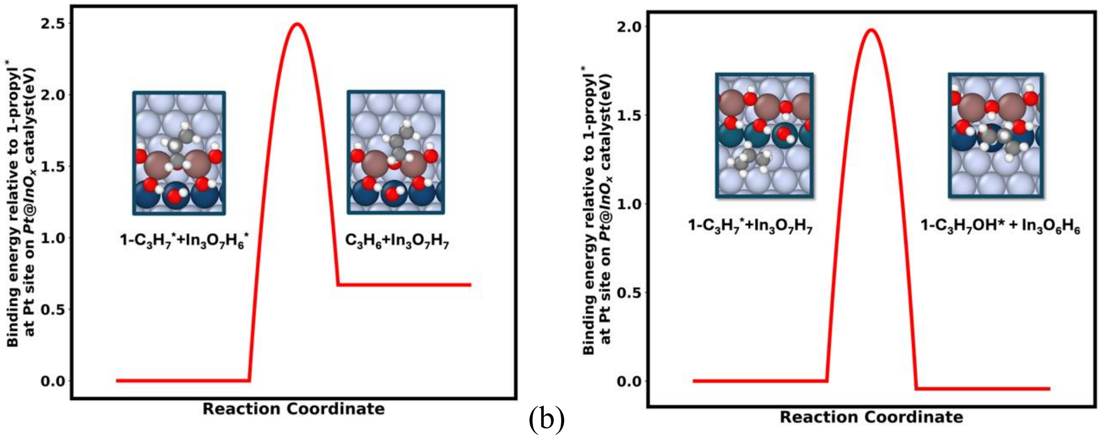

Figure S20: (a) C-H bond activation of 1-propyl* on Pt@InO_xH_y catalyst. (b) C-O bond activation of 1-propyl* at Pt-step in the presence of Pt@InO_xH_y catalyst.

7.7 Potential energy diagram at different OH coverages

Figure S21 shows the potential energy diagram for PDH reaction in the presence of differen O-concentrations in InO_x overlayer structures such as $In_3O_7H_7$ with an In coordination of 3 and $In_3O_3H_3$ with an In coordination of 2. It was found that the early dehydrogenated intermediates such as propane*, 1-propyl*, and propylene* have similar potential energies while the late dehydrogenated intermediates such as 1-propenyl* and propyne* are stable by 0.24 eV and 0.45 eV respectively. From the kinetic barriers, the first C-H activation step of propane* and propylene* dehydrogenation step show kinetic barriers of +0.73 eV and +0.78 eV which are 0.2 eV and 0.1 eV lesser than that on Pt@$In_3O_7H_7$ as shown in Fig. S21. Further, the propylene* desorption barrier is observed as +0.51 eV which is +0.16 eV higher compared to that on Pt@$In_3O_7H_7$. These results show that InO_x surface speciation plays an important role in catalyst reactivity. While the barrier for C-H activation of propane* is low, the higher barrier of propylene desorption, and almost equal barriers for propylene dehydrogenation step reduce propylene selectivity in PDH reaction on Pt@$In_3O_3H_3$ which has lower O-concentration. This shows the importance of O-chemical potential

which correlates with O-concentration in InO_x structure in influencing the catalyst reactivity and stability.

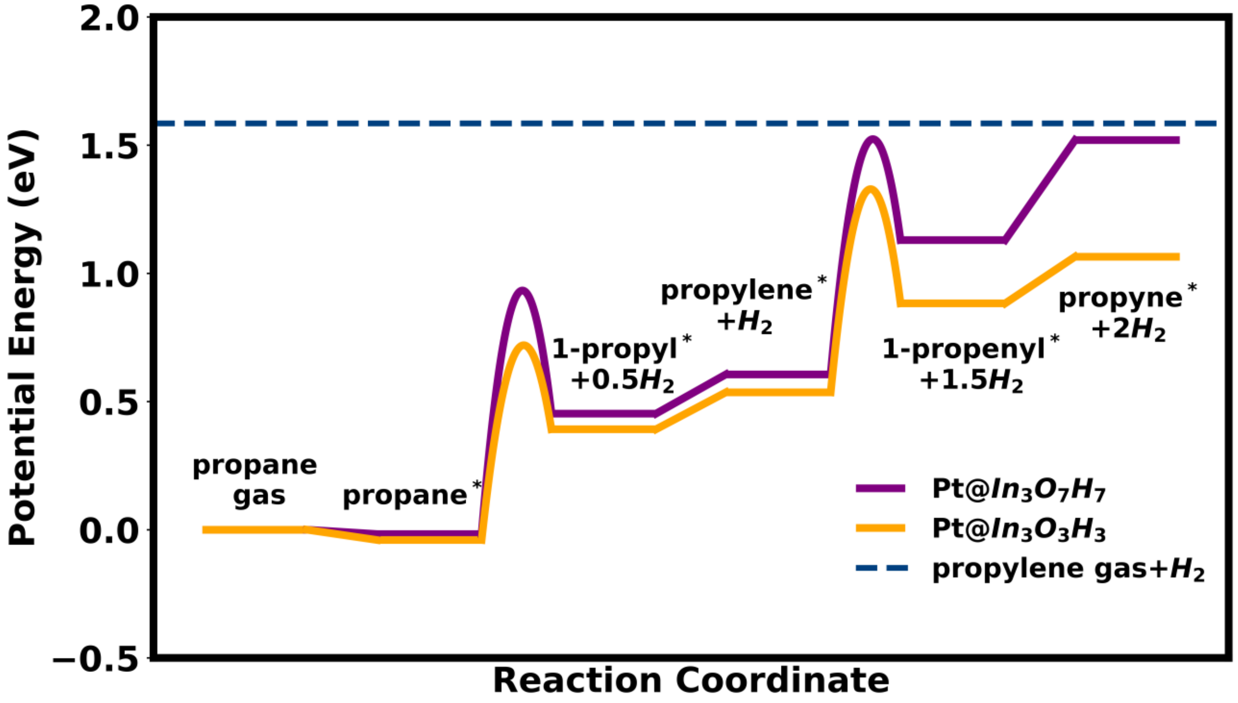

Figure S21: Potential Energy Diagram of dehydrogenation of key reaction intermediates for Propane Dehydrogenation on Pt(322)@$In_3O_7H_7$ and Pt(322)@$In_3O_3H_3$ catalysts.

7.8 Pt lattice constant optimization

Fig. S22 shows the optimized lattice constant obtained as 3.97 Å using the PBE functional as shown in the main text. This is used to create Pt slabs in further calculations.

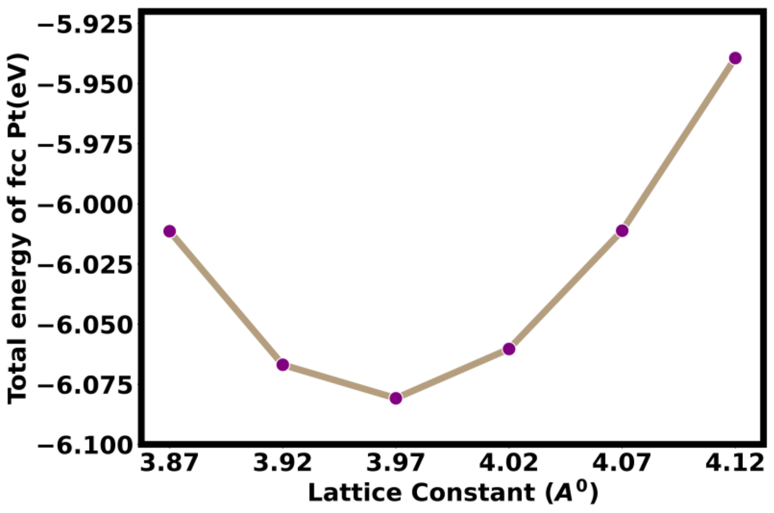

Figure S22: Optimized lattice constant of Pt

7.9 Dipole and Spin Effects

Fig. S23a shows the effect of dipole at different vacuum spacing ranging from 8 to 13 Å. At 11 Å of vacuum spacing on each side of the slab, the difference in energy with and without including dipole effects is close to zero. The spin effects are also found to be negligible in InO_x structures with different O/OH functional groups as shown in Fig. S23b.

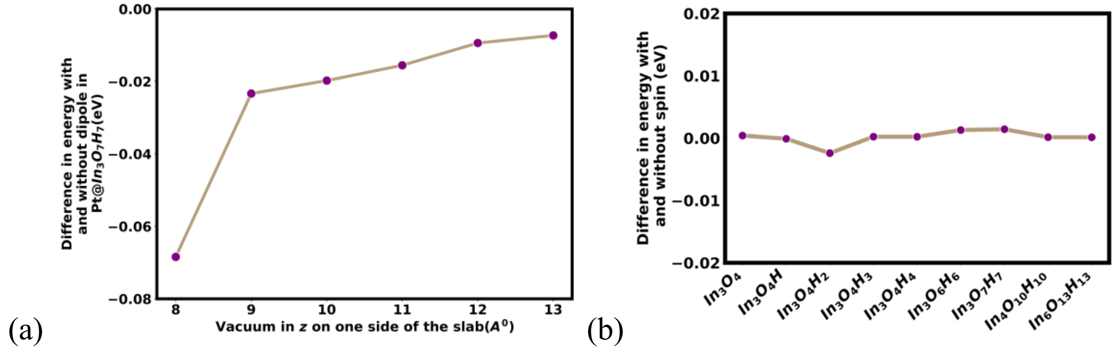

Figure S23: (a) Effect of dipole on vacuum spacing on one side of the slab in Pt-$In_3O_7H_7$ catalytic system. (b) Effect of spin on the binding energy of different overlayer structures with varied coordination and OH coverage.

REFERENCES

(1) Yan, H.; He, K.; Samek, I. A.; Jing, D.; Nanda, M. G.; Stair, P. C.; Notestein, J. M. Tandem In2O3-Pt/Al2O3 catalyst for coupling of propane dehydrogenation to selective H2 combustion. *Science* **2021**, *371* (6535), 1257-1260. DOI: 10.1126/science.abd4441.
(2) Deshpande, S.; Vlachos, D. G. A Data and DFT-Driven Framework for Predicting the Microstructure of Submonolayer Inverse Metal Oxide on Metal Catalysts. *The Journal of Physical Chemistry Letters* **2024**, *15*, 2715-2722. DOI: 10.1021/acs.jpclett.4c00220.
(3) Rizo, R.; Fernández-Vidal, J.; Hardwick, L. J.; Attard, G. A.; Vidal-Iglesias, F. J.; Climent, V.; Herrero, E.; Feliu, J. M. Investigating the presence of adsorbed species on Pt steps at low potentials. *Nature Communications* **2022**, *13* (1). DOI: 10.1038/s41467-022-30241-7.
(4) Deshpande, S.; Maxson, T.; Greeley, J. Graph theory approach to determine configurations of multidentate and high coverage adsorbates for heterogeneous catalysis. *npj Computational Materials* **2020**, *6* (1). DOI: 10.1038/s41524-020-0345-2.
(5) Campbell, C. T.; Sellers, J. R. V. The Entropies of Adsorbed Molecules. *Journal of the American Chemical Society* **2012**, *134* (43). DOI: 10.1021/ja3080117.
(6) Zha, S.; Sun, G.; Wu, T.; Zhao, J.; Zhao, Z.-J.; Gong, J. Identification of Pt-based catalysts for propane dehydrogenation via a probability analysis. *Chemical Science* **2018**, *9* (16). DOI: 10.1039/C8SC00802G.
(7) Seemakurthi, R. R.; Canning, G.; Wu, Z.; Miller, J. T.; Datye, A. K.; Greeley, J. Identification of a selectivity descriptor for propane dehydrogenation through density functional and microkinetic analysis on pure Pd and Pd alloys. *ACS Catalysis* **2021**, *11* (15), 9588-9604. DOI: 10.1021/acscatal.1c01916.
(8) Campbell, C. T.; Sprowl, L. H.; Árnadóttir, L. Equilibrium Constants and Rate Constants for Adsorbates: Two-Dimensional (2D) Ideal Gas, 2D Ideal Lattice Gas, and Ideal Hindered Translator Models. *The Journal of Physical Chemistry C* **2016**, *120* (19). DOI: 10.1021/acs.jpcc.6b00975.
(9) Yang, M.-L.; Zhu, Y.-A.; Fan, C.; Sui, Z.-J.; Chen, D.; Zhou, X.-G. DFT study of propane dehydrogenation on Pt catalyst: effects of step sites. *Physical Chemistry Chemical Physics* **2011**, *13* (8). DOI: 10.1039/C0CP00341G.
